# Starvation-induced metabolic rewiring affects mTORC1 composition in vivo

**DOI:** 10.1038/s41598-024-78873-7

**Published:** 2024-11-16

**Authors:** Edgar Kaade, Simone Mausbach, Nina Erps, Marc Sylvester, Farhad Shakeri, Ron D. Jachimowicz, Volkmar Gieselmann, Melanie Thelen

**Affiliations:** 1 Institute for Biochemistry and Molecular Biology, Medical Faculty, Rheinische Friedrich-Wilhelms-University of Bonn, 53115 Bonn, Germany; 2 Max-Planck Institute for Biology of Ageing, Joseph Stelzmann Str. 9B, 50931 Cologne, Germany; 3 Core Facility Analytical Proteomics, Medical Faculty , Rheinische Friedrich-Wilhelms-University of Bonn, 53115 Bonn, Germany; 4 Institute for Medical Biometry, Informatics and Epidemiology, Medical Faculty, Rheinische Friedrich-Wilhelms-University of Bonn, Venusberg-Campus 1, 53127 Bonn, Germany; 5 Institute for Genomic Statistics and Bioinformatics, Medical Faculty, Rheinische Friedrich-Wilhelms-University of Bonn, Venusberg-Campus 1, 53127 Bonn, Germany; 6 Department I of Internal Medicine, Center for Integrated Oncology Aachen Bonn Cologne Duesseldorf (CIO ABCD), University of Cologne, Cologne, Germany; 7 Cologne Excellence Cluster on Cellular Stress Response in Aging-Associated Diseases, University of Cologne, Cologne, Germany

**Keywords:** Biochemistry, Cell biology, Molecular biology

## Abstract

Lysosomes play a crucial role in metabolic adaptation to starvation, but detailed in vivo studies are scarce. Therefore, we investigated the changes of the proteome of liver lysosomes in mice starved short-term for 6h or long-term for 24h. We verified starvation-induced catabolism by weight loss, ketone body production, drop in blood glucose and an increase of 3-methylhistidine. Deactivation of mTORC1 in vivo after short-term starvation causes a depletion of mTORC1 and the associated Ragulator complex in hepatic lysosomes, resulting in diminished phosphorylation of mTORC1 target proteins. While mTORC1 lysosomal protein levels and activity in liver were restored after long-term starvation, the lysosomal levels of Ragulator remained constantly reduced. To determine whether this mTORC1 activity pattern may be organ-specific, we further investigated the key metabolic organs muscle and brain. mTORC1 inactivation, but not re-activation, occurred in muscle after a starvation of 12 h or longer. In brain, mTORC1 activity remained unchanged during starvation. As mTORC1 deactivation is known to induce autophagy, we further investigated the more than 150 non-lysosomal proteins enriched in the lysosomal fraction upon starvation. Proteasomal, cytosolic and peroxisomal proteins dominated after short-term starvation, while after long-term starvation, mainly proteasomal and mitochondrial proteins accumulated, indicating ordered autophagic protein degradation.

## Introduction

Lysosomes are membrane-limited cellular organelles with an intraluminal acidic pH which is maintained by the vacuolar H^+^-ATPase in combination with various anion- and cation channels^1^. This acidic environment promotes the degradation of proteins, lipids, nucleic acids and polysaccharides by more than 60 known lysosomal hydrolases and their cofactors, followed by an export of metabolites to the cytoplasm by highly specialized transporters, where they are used as substrates for anabolic and catabolic reactions. Since the discovery of lysosomes by Christian de Duve in 1955^2^, a tremendous amount of research has placed the lysosome in the center of a complex signaling network mediating control of cellular metabolism. While the degradative function of the lysosome is carried out inside the lumen, the surface of the lysosome facing the cytosol is covered by protein complexes which participate in intracellular signaling pathways to regulate crucial anabolic and catabolic pathways. In response to alterations of nutrient availability, lysosomes change their size, number, enzymatic activity and positioning (reviewed by^3^). The investigation of the molecular mechanisms underlying these observations uncovered ion and nutrient transporters, protein kinases and phosphatases as well as transcription factors to be part of a lysosomal regulatory network. Central to these findings was the discovery that the localization and activation of the mTOR complex 1 (mTORC1), which contains the crucial kinase mammalian target of rapamycin (mTOR), changes in response to nutrient availability. Under nutrient-rich conditions, mTORC1 binds to the lysosomal surface and is activated. Vice versa, during nutrient starvation, the mTORC1 complex is inactivated and dissociates into the cytoplasm. mTORC1 is composed of its key components mTOR, regulatory-associated protein of mTOR (RAPTOR), mammalian lethal with Sec13 protein 8 (mLST8) and the two inhibitory subunits proline-rich AKT substrate 40 kDa (PRAS40) and DEP-containing mTOR-interacting protein (DEPTOR)^4–6^. It is the center of a large protein supercomplex on the lysosomal surface, additionally containing the RagA/RagC and RagB/RagD GTPase heterodimers and the pentameric Ragulator complex. mTORC1 can be activated by the small GTPase ras-homolog enriched in brain (Rheb)^7^, which itself is inhibited by the tuberous sclerosis complex (TSC). Amino acid-dependent activation is mediated by the GTP/GDP loading state of the RagA/RagC and RagB/RagD GTPase heterodimers, and is further signaled from within the lysosome by the amino acid transporter SLC38A9^8^(for extensive review of mTORC1 activation patterns see^6,9,10^). mTORC1 activity is central to metabolic adaptation. Its activity impacts e.g. transcription of many genes, translation, autophagy, nucleotide biosynthesis, glucose and lipid metabolism.

Mainly fueled by the unraveling of mTORC1 functionality and regulation, there have been considerable advances in the understanding of lysosome-mediated metabolic regulation. While the vast majority of studies addressing starvation-induced effects on lysosomes have been performed *in vitro*^11,12^, there have rarely been studies in animal models^13^, and to our knowledge, no comprehensive dataset on mouse starvation metabolism and lysosomal proteome composition *in vivo* has been published. To gain more insight into the metabolic regulation *in vivo*, we investigated the activity of the mTORC1 complex and changes in body metabolism in C57/Bl6J wildtype mice after short- and long-term starvation. We identified starvation-induced changes of the lysosomal compartment by mass spectrometry comparing lysosome-enriched fractions after 6h and 24h of starvation, and show organ-specific mTORC1 activation patterns and a sequential degradation of organelle-specific proteins by macroautophagy.

## Results

### Starved mice loose body weight and activate ketone body synthesis after prolonged starvation

**Fig. 1:**
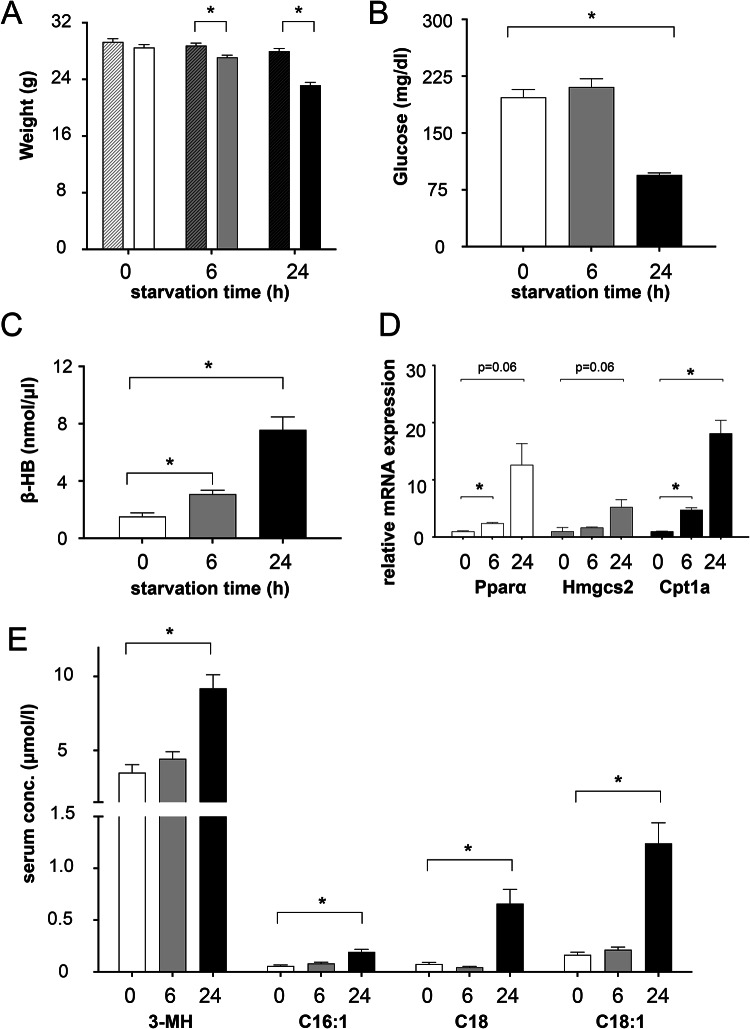
Starvation consequences in mice. 6-month-old male wild-type mice were starved for 6 or 24h. Control mice were fed ad libitum prior to sacrifice and analyses. (**A**) Average body weights are depicted as grams (n = 12–18). Shown are mean + SEM. White bars depict data for the 0h, grey bars for the 6h and black bars for the 24h starved mice. For every time point, striped bars represent the average weight at time of Triton WR1339 injection and solid bars average weight after starvation, at time of sacrifice. (**B**) Blood glucose concentrations (mg/dl) were determined for each group. N = 8–12. Shown are mean + SEM. (**C**) Average concentrations of ketone bodies (nmol/µl), determined by beta-hydroxybutyrate (β-HB) colorimetric assay. N = 8–12. Shown are mean + SD. (**D**) Real-time PCR. Fold changes of ketogenesis-related genes (Pparα, Hmgcs2 and Cpt1a) were analyzed by 2^−ΔΔCt^ method. Samples from control (n = 3), 6h (n = 3) and 24h (n = 4) starved mice were statistically analyzed. Shown are mean + SEM. (**E**) determination of 3-methylhistidine (3-MH) and long-chain acylcarnitines in serum. Shown are mean + SEM. * = p < 0.05.

Responses of cells and organisms to starvation are dependent on the duration of food deprivation. By measurement of body weight, blood glucose levels and ketone body production, we monitored the metabolic adaption to two starvation conditions in mice: short-term starvation for 6h and long-term starvation for 24h. Mice exhibited a significant decrease in body weight with an average weight loss of 3.2% after 6h of starvation progressing to 14.6% after 24h (Fig. 1A). Blood glucose levels did not differ between mice starved short-term and control animals fed *ad libitum*, but dropped from 190 mg/dl to 90 mg/dl after long-term food deprivation (Fig. 1B). In contrast, ketone body production already increased significantly after 6h of starvation and further intensified after 24h as indicated by a twofold and 5.6-fold increase, respectively, of β-hydroxybutyrate in serum (Fig. 1C). When analyzing the gene expression of key ketogenic enzymes, we observed an upregulation of mitochondrial 3-hydroxy-3-methylglutaryl-CoA synthase 2 (Hmgcs2) by 1.7-fold after 6h starvation and 5.2-fold after 24h, and carnitine palmitoyl transferase 1a (Cpt1a) expression levels raised by 4.8-fold and 19.2-fold after short- and long-term starvation, respectively. Both enzymes are involved in fatty acid oxidation and ketogenesis and are regulated by peroxisome proliferator-activated receptor alpha (Pparα), which is activated after prolonged starvation^14,15^ as reflected by a moderate (2.4-fold) to stronger tenfold increase after long-term starvation (Fig. 1D). Due to considerable inter-animal variations, not all differences shown by RT-PCR reached statistical significance. Selected acylcarnitines like C16:1 and C18, which are intermediate metabolites enabling transport of long-chain fatty acids across the mitochondrial membrane, were significantly increased in serum of mice starved for 24h (Fig. 1E). Likewise, 3-methylhistidine as a biomarker of muscle protein degradation in rodents^16^ showed a 2.6-fold increase from 3.5 μM in control mice to 9.2 μM in mice starved long-term (Fig. 1E). In summary, mice of both starvation groups showed clear metabolic adaptations to changed nutrient conditions, with more significant changes in respective metabolic parameters after 24h starvation compared to 6h, respectively. These verified metabolic changes allowed further analysis of lysosome-specific starvation effects.

### Lysosomal protein composition varies depending on starvation time

**Fig. 2:**
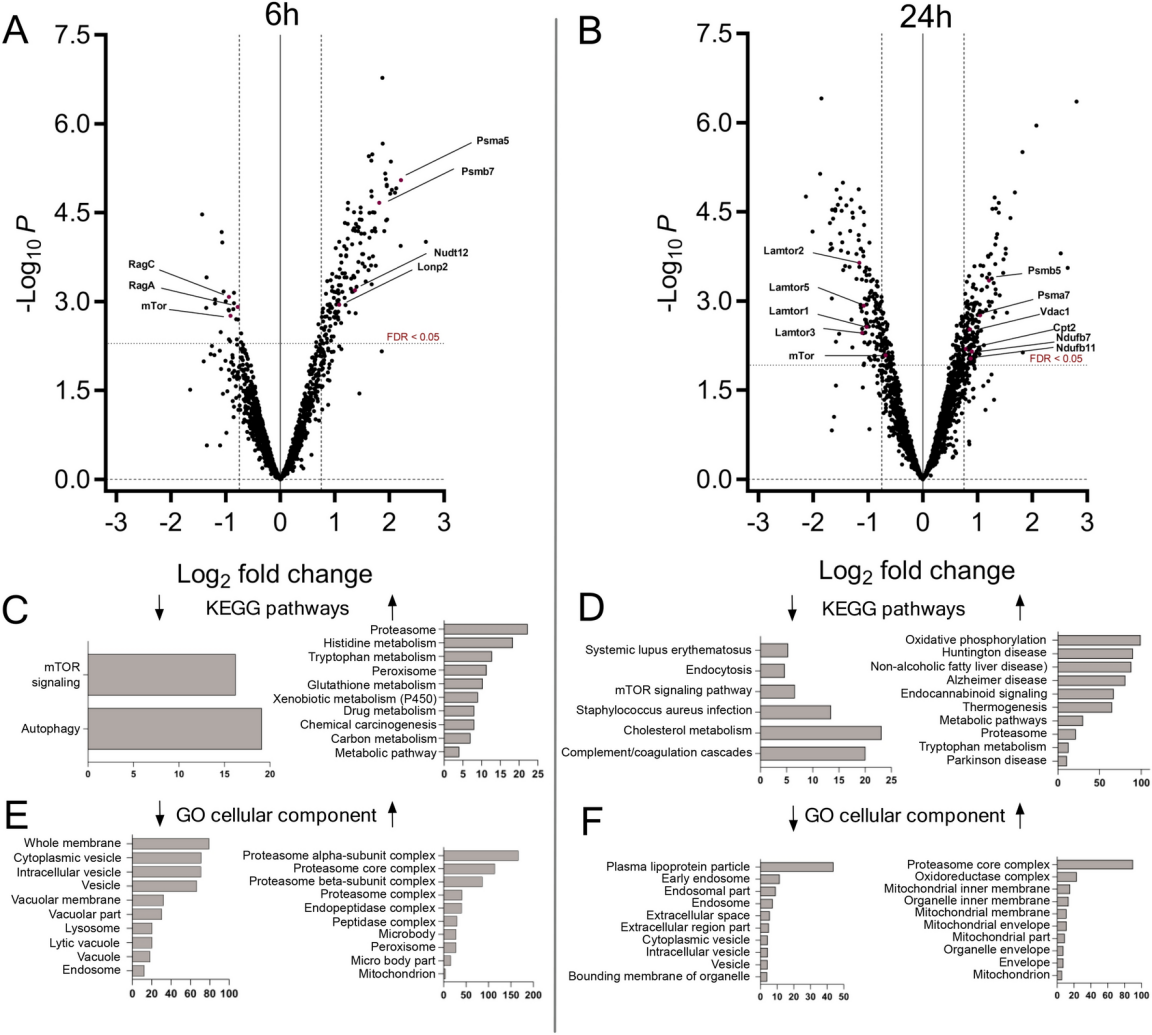
Differentially regulated proteins after short- and longterm starvation. Enriched lysosomal fractions from control, 6h **(A)** and 24h **(B)** (n = 3–4) starved mice were tryptically digested. TMT labeled peptides were mixed and fractionated prior to MS measurement. Raw data were searched by proteome discoverer 2.3. Normalized proteins were statistically analyzed and p-values were corrected by the Benjamini–Hochberg method with a threshold of 0.05. The -log_10_ p-values were plotted against log_2_ fold change of starved/control. Proteins with a log_2_ fold change < -0.75 or > 0.75 are considered differentially regulated. **C-F**: Overrepresentation analysis of KEGG pathways^20–22^ and GO term cellular component with calculated enrichment factors for proteins that were differentially regulated after 6h (**C,E**) or 24 h of starvation (**D,F**), only results with a corrected p-value < 0.05 are shown. In C, D, E, and F left diagrams refer to proteins diminished and right diagrams to proteins enriched in lysosomes, respectively.

Given the pivotal role of the lysosome as a major catabolic organelle and central hub of metabolic regulation, we hypothesized that the observed metabolic alterations in starved mice are accompanied by changes in lysosomal protein composition. Lysosome-enriched fractions isolated from livers of control, 6h and 24h starved mice by differential centrifugation after triton WR1339-induced lysosomal density shift^17–19^ (tritosomes) were subjected to mass spectrometric analysis. Lamp1 immunoblotting demonstrated that the extent of lysosome enrichment was independent of the feeding status (Supplementary Fig. 1), showing a gradual increase in Lamp1 throughout enrichment steps. Proteins of lysosomal fractions were digested with trypsin and tryptic peptides were labeled using a 10-plex tandem mass tag (TMT) labeling, enabling analysis of n = 3–4 biological replicates of 6h and 24h starved animals and controls fed ad libitum in one measurement. Mass spectrometric analysis yielded 3417 identified proteins in total. After filtering low-quality data such as non-unique peptides and single-hit proteins from exported PD 2.3 raw files and only accepting proteins with at least two unique peptides, 1725 proteins were suitable for quantification and further statistical analysis. To enhance the statistical analysis, quality control procedures were applied to investigate the sample distribution and the correlation between replicates. The results were visualized by boxplots and principal component analysis plot (PCA) (Supplementary Fig. 2 and 3), showing a near normal sample distribution. All proteins with a corrected p-value < 0.05 as well as increased or decreased amounts by a log2 fold change of at least -0.75 or 0.75, respectively, according to their starvation/control ratios, were considered elevated or diminished in the lysosomal fraction. Changes of protein abundance in lysosomal fractions (for a complete list, see Table S1) were visualized using Volcano Plots (Fig. 2A and B), depicting data after 6h and 24h of starvation, respectively. Here, it became apparent that the majority of changes in protein amount were due to an elevation of proteins rather than to a diminishment especially after 6h. After 6h and 24h of starvation, respectively, the amount of 155 and 166 proteins was significantly increased including an overlap of 51 proteins (Table S2) for both conditions. In contrast, only 23 and 139 proteins were diminished after 6h and 24h of starvation, respectively. Differently abundant proteins were further analyzed by the WEB-based GEne SeT AnaLysis Toolkit (Webgestalt), analyzing enriched KEGG pathway classifications^20–22^ and GO-terms of cellular components of up- and downregulated proteins (Fig. 2 C-F). The majority of proteins elevated after 6h belonged to the KEGG pathway classifications ‘proteasome’ with a 22-fold enrichment, histidine/tryptophane metabolism (18.3/12.8-fold enrichment) and ‘peroxisome’ (11.4-fold enrichment). After 24h, the pattern of elevated proteins had changed considerably with proteins assigned to ‘mitochondrial oxidative phosphorylation ‘ (99-fold enrichment) being the most abundant followed by KEGG pathway classifications ‘neurodegenerative disorders‘ and ‘metabolic pathways‘. This is also reflected in the GO-term cellular component (CC) classifications, where a mainly mitochondrial cellular localization of proteins enriched after 24h was annotated in 9 of 10 enriched categories (Fig. 2 D). Interestingly, proteins with decreased abundance in tritosome fraction after 6 h (23 proteins) were almost exclusively annotated to the KEGG pathway classifications autophagy (19-fold enrichment) and mTOR signaling pathway (16-fold enrichment). Following the 24-h starvation period, 139 proteins were reduced compared to non-starved animals, here the mTOR-signaling pathway and cholesterol metabolism emerged as significantly enriched pathways. GO analysis of diminished proteins after 24h starvation shows an assignment to mainly lipoproteins and endocytosis proteins (Fig. 2F). In summary, we identified a particular signature of proteins with changed abundances clearly indicating adaptations of the mTORC1 signaling pathway and autophagic protein degradation, which were only partially overlapping between short- and long-term starvation. This suggests differential metabolic responses depending on the duration of nutrient deprivation.

### Changes in bona fide lysosomal proteins are mostly connected to mTORC1 signaling

In lysosomal fractions, proteins usually comprise a mixture of *bona fide* lysosomal proteins with their primary and functional location being the lysosome, and proteins usually resident in other cellular compartments that have been targeted to the lysosome for degradation or temporary location. Therefore, we examined lysosomal and non-lysosomal proteins separately.

In our dataset we quantified 233 proteins which are lysosomal according to the protein center database and gene ontology (GO) analysis tool in PD 2.3, which equals 13.5% of all quantified proteins (listed in table S3). After 6h of starvation, 11 lysosomal proteins were significantly reduced and only 1 lysosomal protein, peroxiredoxin-6, elevated (Tables S1 and S4). Four out of 11 depleted proteins are lysosome-associated proteins related to the mTOR signaling pathway, namely GATOR complex protein WDR59, the Rag complex members RagA and RagC and mTORC1 itself. Of the remaining 7 diminished proteins assigned to lysosomes via GO analysis, only three—all membrane proteins—can be considered as resident lysosomal proteins, namely WD repeat containing protein 81 (Wdr81), transmembrane protein 106B (Tmem106B), and ganglioside- induced differentiation-associated protein 2 (Gdap2). After 24h of starvation, 17 lysosomal annotated proteins were reduced and only peroxiredoxin-6 elevated (Tables S1 and S4, respectively). Eight out of 17 reduced lysosomal proteins are lysosome-associated proteins involved in the mTORC1 signaling pathway: the Ragulator complex components Lamtor1, 2, 3 and 5 and the GATOR complex subunits WDR59, MIOS, NPRL2 and DEPDC5. Of the remaining 9 proteins assigned to the lysosome by GO analysis again only three can be considered as primarily lysosomal: Wdr81, Gdap2 and ATP-binding cassette sub-family A member 2 which, however, also occurs in endosomes. For a detailed listing of all results including fold-changes please refer to tables S1-S4.

In conclusion, under starvation conditions, the quantities of proteins associated with lysosomes, as determined by GO analysis, were predominantly reduced, with a particular emphasis on the depletion of mTORC1-related proteins.

**Fig. 3:**
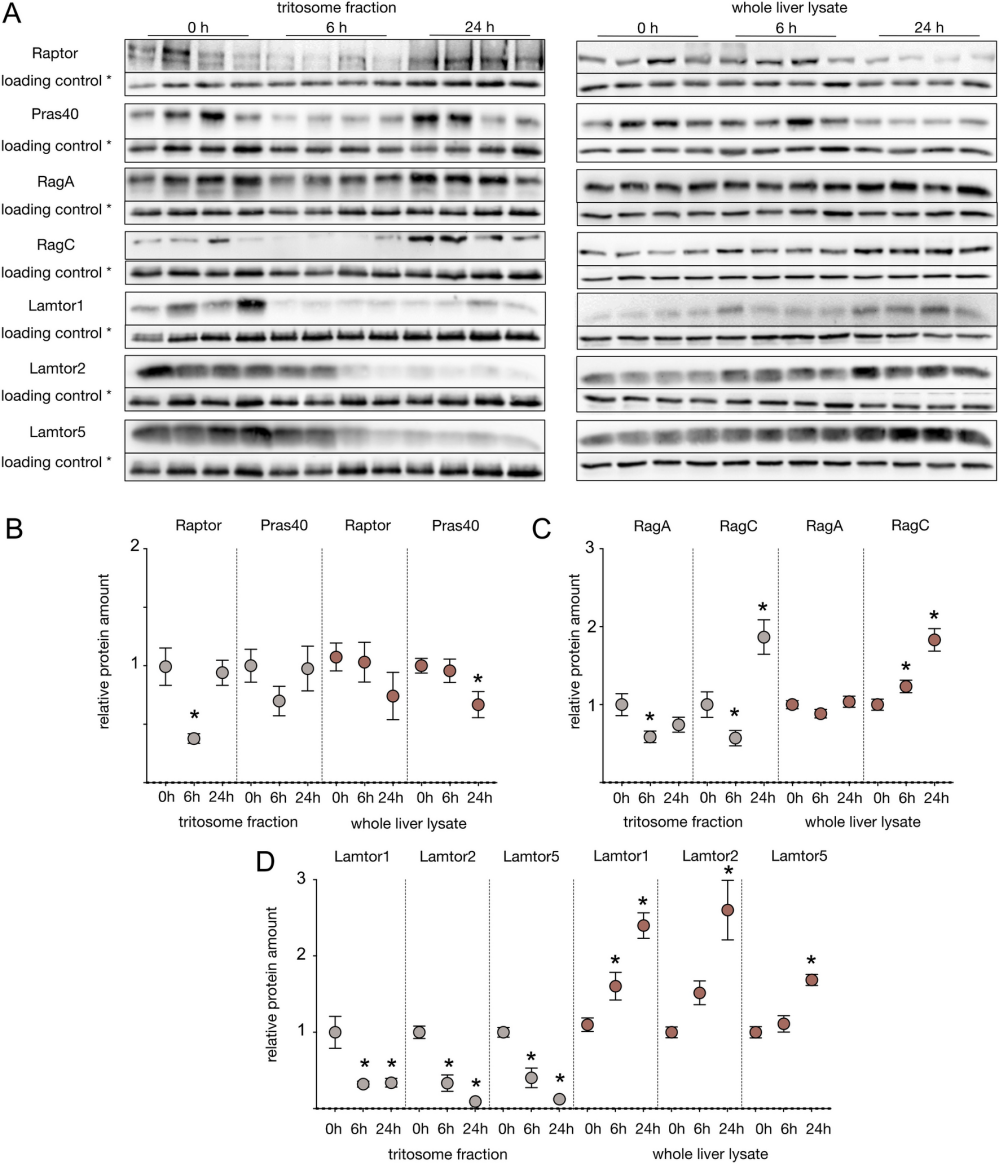
Regulation of mTORC1 complex proteins. (**A**) Enriched tritosome fractions and whole liver lysates were extracted from mice starved for six hours or one day and control animals fed ad libitum before sacrifice. Proteins were separated by SDS-PAGE, blotted and probed with indicated antibodies.* = loading controls were Gapdh for whole liver lysate and tripeptidyl peptidase 1 (Tpp1) for tritosome fractions. (**B-D)** Densitometric quantified signals were normalized to the corresponding Gapdh/Tpp1 intensity. Shown are mean + SEM; n = 8. * = p < 0.05. Control samples were set to 1. Western blot images were cropped for better representation. Full-size images are available in the supplementary information.

The observed nutrient dependence of components of the mTORC1 complex on the lysosomal surface has been described in multiple cell culture models, e.g. HEK 293T cells^23^. Nutrient availability causes mTORC1 association with the cytosolic lysosomal surface, while starvation causes its dissociation into the cytoplasm. This is in accordance with the lysosomal mTORC1 diminishment upon starvation in our mass spectrometric data set. However, we wanted to confirm these data by western blot analyses. Since we were unable to reliably detect mTOR directly by western blots, we used antibodies against RAPTOR and PRAS40, which are integral components of the mTORC1 complex to monitor its localization. To verify that changes in protein amounts detected in tritosome fractions were not due to an overall change in protein amount, we quantified all proteins selected for western blot analysis in tritosome fractions and whole liver lysates with respect to Gapdh in lysates and T1 in tritosomes, respectively (Fig. 3). While Raptor levels in crude liver lysates of animals starved for 6h and 24h did not show any significant changes, in tritosome fractions from the same animals, Raptor was significantly diminished to around 40% after 6h of starvation and reached baseline levels again after 24h. Pras40 showed a comparable pattern (Fig. 3 A, B). Essential recruiters of mTORC1 to the lysosomal surface are the Rag GTPase heterodimers, part of the Ragulator-Rag complex, which bind to mTORC1 in their active forms, GTP bound-RagA/B and GDP bound-RagC/D (reviewed by^9^). Our mass spectrometric data showed RagA to be significantly diminished by a log2 fold change of -0.78 after 6h, but not after 24h of food deprivation, which was consistent with the western blot analysis (Fig. 3). Meanwhile, in mass spectrometry RagC showed a log2 fold change of -0.94 after 6h and -0.6 after 24h. In western blot analysis, reduction of RagC protein amount after 6h was verified however, after 24h, an increased protein amount compared to baseline samples was observed (Fig. 3A, C). Except for the latter finding, changes of mTORC1 complex members were therefore in accordance with mass spectrometric data.

The pentameric Ragulator complex is located on the lysosomal surface and plays an important role in mTORC1 activation^9^. The binding of both RagA/B and and RagC/D heterodimers to the lysosomal surface is mediated by Ragulator which consists of the proteins Lamtor1-5. No dissociation of Lamtor proteins from the lysosome has been reported in the literature so far, however, log_2_ fold change ratios of Lamtor1, 2, 3 and 5 in our mass spectrometric results indicated indicated reduced amounts after 6h and further reduction after 24h (Table S1 and S2).

Western blot validation experiments confirmed a significant decrease in the abundances of Lamtor1, 2 and 5 in tritosomes after long-term- and also short-term starvation. At the same time, the analysis of all three Ragulator complex proteins in crude liver lysate revealed a continuous increase in Ragulator protein amount during the starvation period. Thus, overall, our mass spectrometric and western blot data are in accordance with a dissociation of the Ragulator complex from the lysosomal surface into the cytoplasm.

### Differential mTORC1 responses to starvation: Deactivation followed by reactivation in liver, continuous deactivation in muscle and unchanged activity in brain

**Fig. 4:**
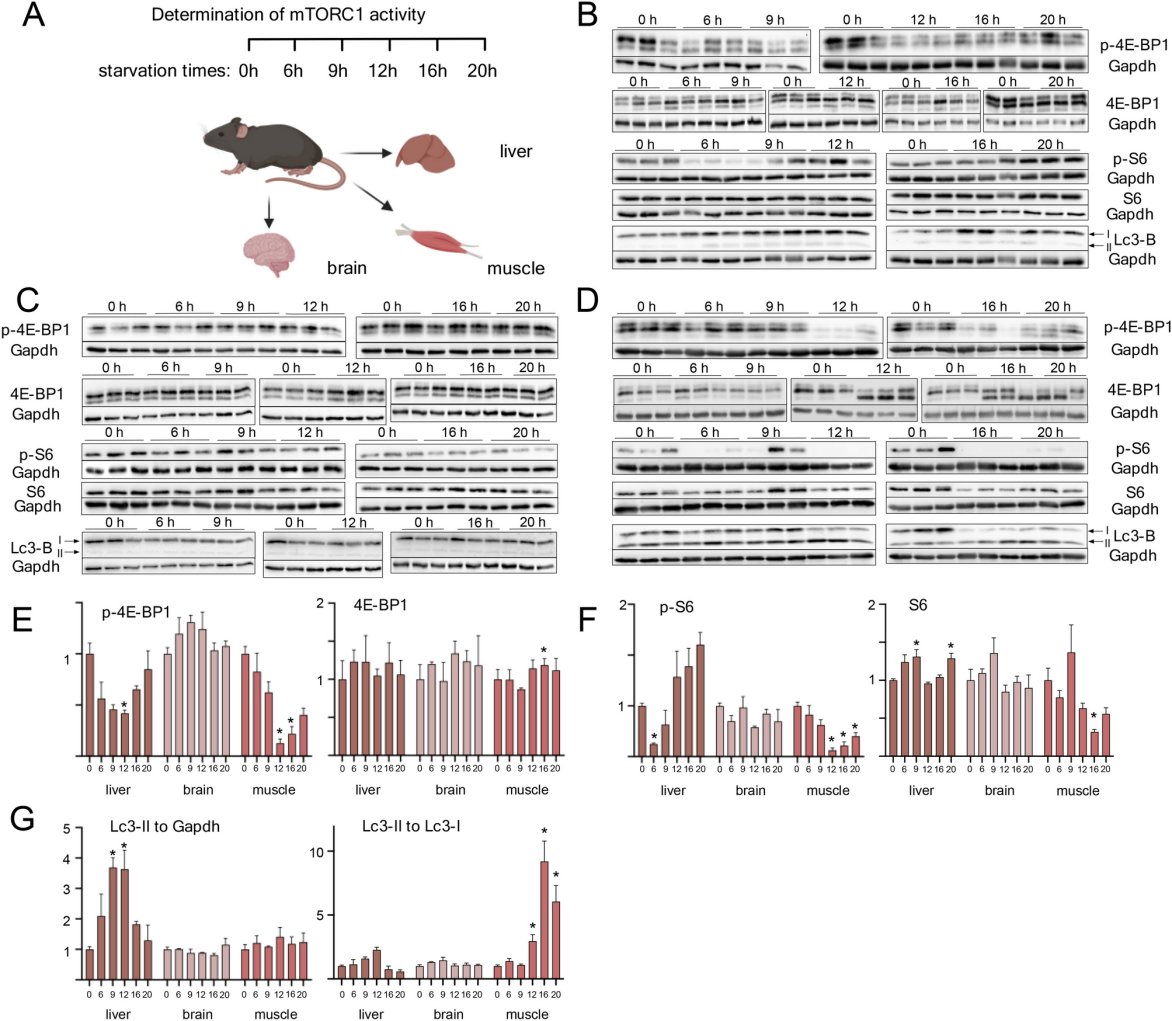
mTORC1 activity during starvation time course. (**A)** Schematic overview about experimental flow. Created in BioRender. Thelen, M. (2023) BioRender.com/c65y449. Adult C57BL/6 mice were starved for 6h, 9h, 12h, 16h or 20h and control mice were *fed ad libitum* before sacrifice. After liver **(B)**, brain **(C)** and muscle **(D)** were removed and lysed, proteins were separated by SDS-PAGE, blotted and probed with indicated antibodies ( **E**–**F**) Densitometric quantified signals were normalized to the corresponding Gapdh intensity. G: LC3-II signals were either normalized to Gapdh (left diagram) or LC3-I (right diagram). Respective controls were set to 1. Shown are mean + SEM; n = 3. * = p < 0.05. Western blot images were cropped for better representation. Full-size images are available in the supplementary information.

When bound to the lysosomal surface during nutrient availability, mTORC1 is active and phosphorylates specific downstream targets. To investigate how mTORC1 activity correlates with the reduction of mTORC1 components in tritosome fractions, we analyzed the phosphorylation status of mTORC1 target proteins during short- and long-term starvation (Fig. 4). Importantly, while starvation times for investigations of mTORC1 activity in vitro mostly vary from several minutes to one hour, no conclusive data on mTORC1 activity in vivo have been reported. Wildtype mice were subjected to food starvation between 6 and 20h and the extent of phosphorylation of the mTORC1 targets 4EP-binding protein (4E-BP1) and S6 kinase (S6K) was determined. Since the metabolic adaptation to catabolism is not limited to liver but also affects muscle and brain, these organs were also included into the analysis to generate a comprehensive picture of mTORC1 activity in vivo (Fig. 4).

In liver, a continuous decrease in phosphorylation of 4E-BP1 was observed after 6, 9 and 12 h of starvation, followed by an increase and reaching of control levels after 16 and 20h, respectively (Fig. 4B,E). Similarly, S6 phosphorylation dropped to minimum levels already after 6 h of starvation and even surpassed initial levels already after 12 h of starvation (Fig. 4B,E). This argues for a rapid mTORC1 reactivation in liver after initiation of starvation. The total levels of 4E-BP1 and S6K were unchanged during the starvation time course, except for a small but significant increase in S6K protein levels after 6 and 20h of starvation (Fig. 4B, E–G). Since the activation of autophagy is tightly regulated by mTORC1 and starvation-induced autophagy indicates mTORC1 inactivation^24^, autophagy was monitored by LC3B immunoblotting, following the ratios between the lipidated (LC3II) and non-lipidated forms (LC3I) of LC3B, either relative to Gapdh levels or to each other (Fig. 4G). This ratio shifted to the lipidated form II of LC3B in liver tissue after 9 and 12 h of starvation, inversely correlating with mTORC1 target protein phosphorylation (Fig. 4 B, G). p4E-BP1 in skeletal muscle decreased to around 15% of control levels only after 12 h of starvation, and although there seems to be an increasing pattern similar to liver, levels of phosphorylated protein stay well below control levels. A similar pattern was found for S6 phosphorylation. Thus, in contrast to liver muscle does not show a pronounced reactivation of mTORC1 after prolonged starvation, which may warrant a more in-depth analysis. While LC3-II levels remained unchanged in muscle compared to loading control, LC3-II/LCI levels strongly increased (Fig. 4G) indicative of increased autophagic flux. In brain, no changes of phosphorylation or proteins levels were observed for any of the investigated proteins (Fig. 4D-G). In conclusion, our data show an organ specific mTORC1 activation pattern in liver, muscle and brain. While in liver, mTORC1 is deactivated early after 6h of starvation followed by a rapid reactivation, this is not true for muscle, where mTORC1 is deactivated later and without reactivation after prolonged starvation periods. No change of mTORC1 activity or LC3I/LC3-II levels was detected in brain.

### Changes in non-lysosomal proteins indicate ordered protein degradation during starvation

**Fig. 5:**
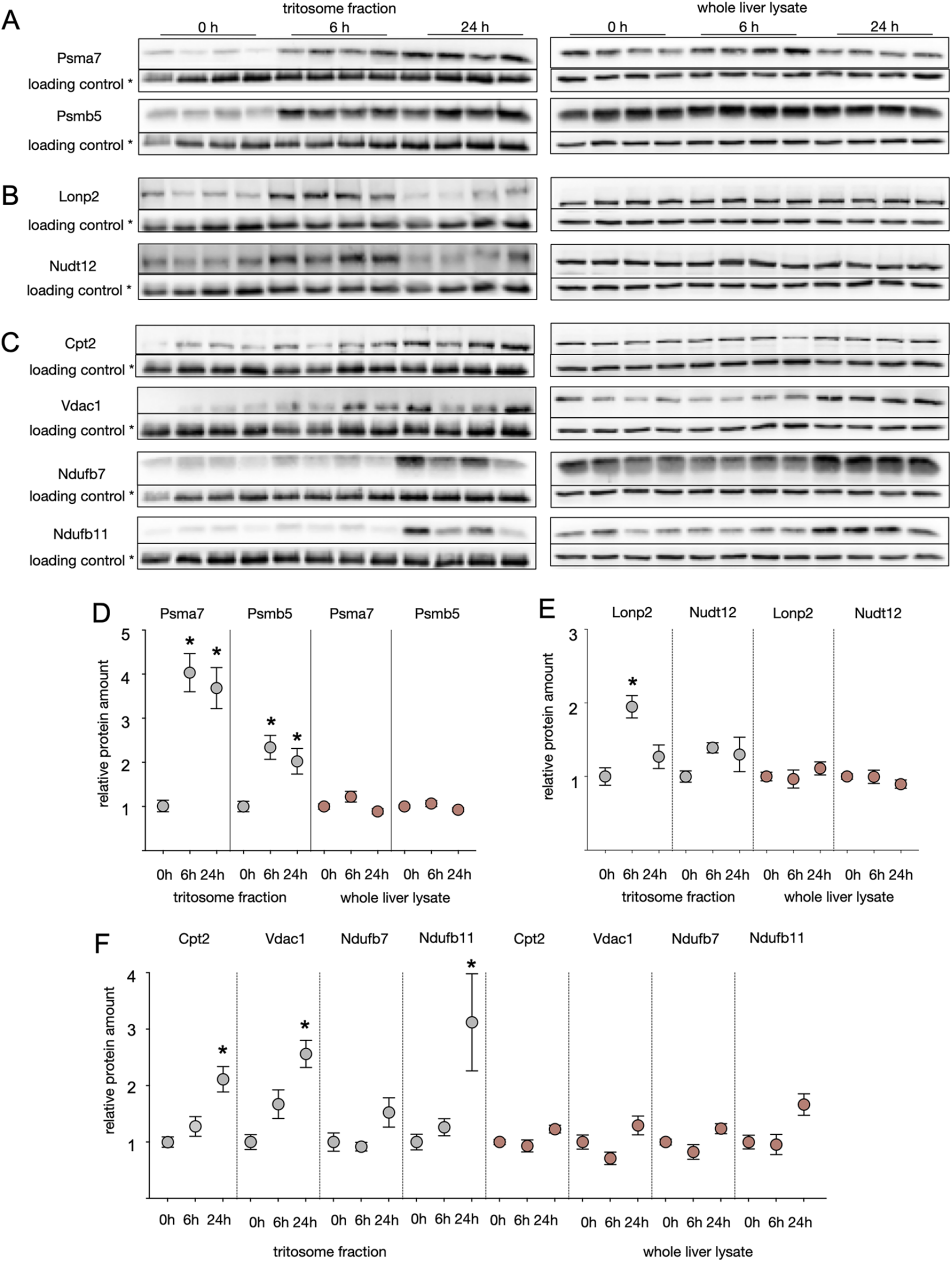
Accumulation of proteins from selected organelles indicate autophagy. Whole liver lysates and enriched trisosome fractions were extracted from mice starved for six hours or one day and control animals fed ad libitum before sacrifice. Proteins annotated to the proteasome **(A)**, peroxisome **(B)** or mitochondrium **(C)** were separated by SDS-PAGE, blotted and probed with indicated antibodies.* = loading controls were Gapdh for whole liver lysate and tripeptidyl peptidase 1 (Tpp1) for tritosome fractions. (**D-F)** Densitometric quantified signals were normalized to the corresponding Gapdh/Tpp1 intensity. Shown are mean + SEM; n = 8. * = p < 0.05. Control samples were set to 1. Western blot images were cropped for better representation. Full-size images are available in the supplementary information.

An increased presence of non-lysosomal proteins in the tritosomal fractions can be either caused by their enhanced association to the outer lysosomal membrane, or an accumulation inside the lysosomal lumen due to autophagy and indicative of degradation. The degradation of various organelles by macroautophagy was reported in several publications (reviewed by^25^). For further analysis, we concentrated on selected proteins showing with increased amounts in tritosome fractions based on mass spectrometry and verified their lysosomal accumulation by western blotting. After 6h and 24h of starvation, 14 and 12 proteasomal subunits, respectively, were shown to be enriched in tritosomal fractions according to mass spectrometry (Table S1). We confirmed this result through western blotting, revealing a fourfold lysosomal increase of Psma7 and a 2.5-fold increase of Psmb5 (Fig. 5A, D). In contrast, the Psma7 and Psmb5 levels in whole liver lysates remained unchanged (Fig. 5A, D). KEGG pathway and GO analysis of proteins upregulated after 6h further revealed strong enrichment of peroxisomal proteins in our dataset (Fig. 2 C,E), for example of peroxisomal trans-2-enoyl CoA reductase and phytanoyl-CoA dioxygenase, that are enriched after 6 but not 24 h of starvation (Table S1). The peroxisomal proteins Lon peptidase 2 (Lonp2) and NAD-capped RNA hydrolase (Nudt12), were probed by western blotting and while Lonp2 shows increased protein levels of 1.9 after 6h but not 24h of starvation, a slight increase in Nudt12 did not reach significance. Protein levels in whole liver lysates remained constant (Fig. 5B, E). As detailed above, after 24h of starvation, GO-term analysis indicated a strong enrichment of mitochondrial proteins within tritosome fractions (Fig. 2 D, F). Carnithine-palmitoyltransferase 2 (Cpt2), voltage-dependent anion-selective channel 1 (Vdac1), the NADH dehydrogenase subunits Ndufb11 and Ndufb7 were significantly increased in tritosomes according to mass spectrometry (Table S1). Western blot confirmed that Cpt2, Vdac1 and Ndufb11 are elevated in tritosomes in particular after 24h of starvation and less so at 6h (Fig. 5 C; F), respectively, while Ndufb7 protein level changes were statistically significant only in our mass spectrometry analysis but not in western blotting (Fig. 5 C, F). Thus, based on our mass spectrometry data and western blot analysis, an ordered degradation pattern of proteins within the lysosome emerges: proteasomal and peroxisomal proteins undergo early degradation during starvation, followed by the enhanced degradation of mitochondrial proteins in later stages.

## Discussion

The current study provides a comprehensive, lysosome-focused analysis of starvation metabolism in mice. Starvation induces strong metabolic changes on an organismal and cellular level. We were able to measure these changes by detecting a weight loss of 3.2% and 14.6% after 6h and 24h, as well as a decrease in blood glucose levels after 24h but not 6h of starvation. The observed weight loss and drop in blood glucose levels are within the range reported in other studies, as reviewed by Jensen and colleagues^26^, verifying mice in our study displayed a robust induction of starvation metabolism and different starvation times were reflected by specific metabolic changes.

After consumption of stored glycogen in the liver, gluconeogenesis is used for glucose production from glucogenic amino acids. Source of these amino acids can be breakdown of intracellular proteins by autophagy, which is essential to maintain blood glucose levels during starvation periods^27^. Early studies have shown that during a 48 h starvation time, 7-weeks old CD-1 mice lose on average 36% of their hepatic protein content with a more pronounced decline in the initial 12 hours^28^. In our study high levels of 3-methylhistidine at 24h of starvation as a marker of muscle protein degradation correlated with the decline of blood glucose suggesting that this low glucose level is maintained by gluconeogenesis from muscle derived glucogenic amino acids. In agreement with this mTORC1 activity in muscle remained unchanged at 6h and was reduced only after 12h of starvation. Concomitantly LC3-I levels declined as an indicator of autophagy. The degradation of proteins by autophagy is generally divided into non-selective bulk autophagy, thought to occur in response to nutrient deprivation and precluded by ubiquitination of cargo or selective autophagy via specific receptor proteins^25^. Our analysis indicates varying kinetics of protein accumulation in the lysosomal compartment, suggesting that protein degradation due to starvation in vivo follows a predetermined sequence. Previously, a mass spectrometry-based study of amino acid starvation in vitro defined six distinct protein clusters based on their cellular localization. This analysis of the whole cell proteome revealed a sequential degradation pattern of proteins, starting with cytosolic proteins and proteasomes later followed by mitochondrial proteins^29^. This finding aligns with research by Cuervo and co-workers, who observed the accumulation of proteasomes in rat liver lysosomes after nutrient starvation^30^, and studies on HeLa cervical carcinoma cells demonstrated that starvation-induced autophagy by amino acid depletion leads to a rise in autophagic proteasome degradation after its ubiquitination^31^. These results are in accordance with our data which show a degradation of proteasomal proteins after 6h and mitochondrial proteins after 24h of starvation, respectively. We further see indications of a preferential degradation of peroxisomal proteins after 6 but not 24h, which needs further experimental validation. Mitochondria are thought to be excluded from bulk autophagy mechanisms in early starvation periods by morphological adaptations in vitro and in vivo^32^and may be degraded at later timepoints by specific mitophagy, although we did not find explicit accumulation of known mitophagy adaptor proteins^25^.

Nutrient deprivation further induces ketone production to provide an alternative fuel source for the brain. This was reflected in our starved mice by increased beta-hydroxybutyrate levels and increased transcription of ketogenesis-related genes. The observed increase in ketogenesis is consistent with the inactivation of mTORC1 as displayed by dissociation of mTORC1 from the lysosomal compartment after 6 h of starvation and decreased phosphorylation of 4E-BP1 and S6 kinase. mTORC1 activity has been shown to control the production of ketone bodies in vitro and in vivo by a liver-specific knock-out of Tsc1 in mice, which leads to defective ketogenesis^33^. How the reactivation of mTOR that we observed in liver correlates with ongoing ketogenesis remains to be investigated further. Regarding the composition of the mTORC complex during the different starvation phases, we detected localization changes of the TORC1 complex members Raptor and Pras40. Our data suggest a dissociation of mTORC1 from the lysosomal surface after 6h and a reassociation after 24h in liver. This correlates with mTORC1 target protein phosphorylation status which is low after 6h of starvation and increases again upon longer starvation. This indicates an early inactivation of mTORC1 correlating with its dissociation from the lysosomal surface, followed by retranslocation and reactivation. mTORC1 activation and lysosomal localization requires binding of Ras-related small G-proteins, Rag- and Rheb GTPase, in their active form (reviewed by^34^). The Ragulator complex, consisting of the proteins Lamtor 1–5, is an activation platform on the lysosomal surface interacting with a variety of proteins^35^and has been described to anchor mTORC1 to the lysosomal surface via interaction with the Rag GTPases^11^. Furthermore, it signals amino acid availability to mTORC1 and interacts with SLC38A9 and the v-ATPase^35^. While the lysosomal presence of the Rag GTPases analogous to mTORC1 complex members is reflected in our data, we observed decreasing levels of the Ragulator proteins Lamtor 1, 2, 3 and 5. The Ragulator complex is involved in starvation responses in vitro^36^, but it has never been reported that it may change its localization or detach from the lysosome. A possible alternative mechanism of mTORC1 reactivation without Ragulator involvement in vivo, as suggested by our observations, has not yet been described. It requires further clarification that lays beyond the scope of this present study. In vitro, mTORC1 reactivation during starvation conditions has been observed in different cell lines and the underlying mechanism is not completely understood. While Yu and colleagues showed knock-out of ATG5 or ATG7 reduces reactivation of mTOR after starvation in rat kidney cells in vitro, suggesting reactivation is mediated by nutrients gained by autophagic degradation^37^, another study by Buel et al. found no such effect of ATG5 knock-out but instead demonstrated the Pi3K/Akt pathway to be responsible for mTORC1 reactivation in U2OS cells^38^. A study using the liver cancer cell lines Huh-7 and HepG2 also proposed Akt activation during starvation to mediate mTORC1 activity^39^.

After long-term starvation, protein degradation especially in muscle could be detected by an increase in 3-methylhistidine levels. In contrast to liver tissue, in muscle no reactivation of mTORC1 could be detected which is in accordance with the continuously elevated 3-methyl-histidine levels as an indicator of ongoing protein degradation. This can be reconciled with the result that mice with muscle-specific deletion of mTOR or Raptor display muscle atrophy^40,41^. Overall, the observed metabolic changes are in line with the general perception of starvation metabolism, with the liver mediating the nutrient supply of the body by stabilizing blood glucose levels^27^and producing ketone bodies. The substrates for these processes do not only stem from liver proteins, but are yielded from muscle through release of amino acids from protein degradation by e.g. autophagy, that are used for gluconeogenesis. Expectedly, mTORC1 activity in brain was not affected by starvation conditions and showed no changes in comparison to control mice, since here a permanent nutrient supply is ensured by glucose transporter 3 expression and the uptake of ketone bodies produced by the liver^42,43^.

Technically, the isolation of tritosomes from mouse liver enables a reliable purification of lysosomal fractions from our study mice, as treatment of mice with a Triton-based detergent leads to a lysosomal density shift^44^. This density shift is accompanied by changes in the tritosomal lipid composition. In an earlier study, we have compared tritosomes with a more crudely enriched 20,000 × g fraction, showing that changes in protein amounts between tritosomes from wildtype and Cln6-deficient mice, which display a lysosomal storage disorder, could be reproduced in lysosomal fractions devoid of Triton WR1339^45^. Most importantly, due to our control group which has been treated equally to the fasted animals, we believe that the changes detected in this study are truly caused by fasting and not by Triton WR1339 treatment. The total protein number as well as the amount of lysosomal proteins identified in our analysis is comparable to similar studies^46,47^and normalized replicates showed a close to normal distribution and high correlation of between replicates of the same nutrient condition, demonstrating the validity of our approach. Our mass spectrometric dataset shows the presence of various proteins from basically all cellular compartments. Very likely, most of these proteins are present within lysosomes to be degraded, but we cannot exclude a contamination with small amounts of other cellular organelles, although they typically sediment at densities different from the lysosome-containing interphase band within the gradients^17^.

In conclusion, our study demonstrates novel insights to cellular metabolism and signaling during nutrient deprivation. Accumulation of distinct proteins at both starvation time points indicated an ordered degradation scheme during nutrient deprivation. We could show *in vivo* that in liver, mTORC1 is reactivated after prolonged starvation time by an unknown mechanism that may be independent of the Ragulator complex. In skeletal muscle, mTORC1 shows a gradual deactivation, while it remains continuously activated in brain tissue.

## Materials and methods

### Animals

Mice were housed according to the respective institutional guidelines in the animal facility at the University Hospital Bonn, and experimental procedures were performed according to the institutional guidelines and approved by the Landesamt fuer Natur, Umwelt- und Verbraucherschutz Nordrhein-Westfalen with the application number 84–02.04.2017.A142. All experiments were conducted in compliance with the ARRIVE guidelines. All mice used in this study belonged to the C57/Bl6J substrain and were bred in-house or purchased from Charles River. For starvation, mice were deprived of food for 6, 9, 12, 16, 20 or 24 h while water was available *ad libitum*. Euthanasia was performed by cervical dislocation.

### Lysosome enrichment from mouse liver

Lysosomes were isolated from mouse livers using a sucrose-gradient-based-technique^44^as described previously^46^. Briefly, 3 days ahead of sacrifice, adult mice were intraperitoneally injected with 17% triton WR1339 solution (Sigma-Aldrich, St. Louis, MO) in 0.9% NaCl at 4µl per gram of body weight. After extraction, the liver was homogenized with 3 strokes in 5 volumes of 250 mM sucrose on ice using a Teflon Potter Elvehjem homogenizer. After differential centrifugation, a pellet containing mitochondria and lysosomes (ML), was resuspended in sucrose forming the first layer of a discontinuous sucrose gradient. After ultracentrifugation, a lysosome-containing interphase band was collected, stored at -20 °C and from here on referred to as “tritosomes”.

### Sample preparation for mass spectrometry and TMT labeling

Cell lysates were digested in-solution on centrifugal filter units adapted according to^48–51^. In brief, solutions of 50 µg protein were loaded onto 10 kDa cut-off modified PES centrifugal filters (Pall Filtersystems, Crailsheim, Germany) and reduced with 20 mM DTT at 55°C for 30 min. 40 mM acrylamide was used for alkylation of thiol groups for 30 min at RT. After buffer exchange to 20 mM tetraethylammoniumbromide (TEAB), 0.5% natriumdesoxycholate (SDC) in a total volume of 50 µl, digestion of protein with a 1:100 trypsin:sample ratio was performed for 10 h at 37°C. Peptides were collected and SDC was precipitated with TFA (2% final). Remaining SDC was removed by phase transfer with equal volume of ethyl acetate. Peptides were vacuum concentrated, redissolved in 20 mM TEAB, and labeled with isobaric TMT10plex reagents (Thermo Fisher Scientific, Darmstadt, Germany). Redissolved and pooled peptides were desalted on Oasis HLB cartridges (Waters GmbH, Eschborn, Germany). Eluates containing 70% acetonitrile, 0.1% formic acid (FA) were dried and fractionated to 12 fractions according to their isoelectric point with an Offgel fractionator (Agilent Technologies, Waldbronn, Germany). Fractions were vacuum concentrated, dissolved in 20 µl 5% ACN, 5% FA and desalted using C18 homemade STAGE tips as described^52^.

### LC–MS measurements and data analysis

Measurement of mass spectrometry samples was performed as extensively described in^45^(Orbitrap Fusion Lumos, SPS-MS3 measurements). MS raw files were analyzed by the proteome discoverer software 2.3. For peptide identification, database searches were performed using an in-house made server (Mascot 2.6.1). Using the annotated protein sequences (SwissProt, Mus muscululs sequencs) and contaminants of the common repository of adventitious proteins (cRAP) as reference databases. A maximum of two trypsin missed cleavages was accepted. The precursor mass tolerance was 10 ppm and the fragmentation tolerance (CID) was 0.5 Da. Propionamide at cysteine, TMT on N-terminus and lysine were searched as static modifications, oxidation at methionine was set as dynamic modification. The percolator algorithm^53^ was applied to validate the Mascot results. Spectral matches with a q-value < 0.01 were sent to a second Mascot database search of semi-tryptic peptides with one missed cleavage. Precursor and fragment mass tolerance parameters were kept the same. In addition to the previously mentioned dynamic modifications, acetylation at protein N‐termini was added. Quantified reporter ions were derived from the MS3 level. Peptide spectral matches (PSMs) were filtered for a strict FDR of 0.01 and ‘total peptide amount’ was used as a normalization method.

### Statistical analysis

Raw MS-data, processed by Proteome Discoverer 2.3, were subjected to a comparative statistical analysis. The statistical analyses of the peptide-spectrum match (PSM) level data were carried out in R environment (R version 4.3.3)^54^using an in-house developed workflow. The R-script was provided by Dr. Farhad Shakeri from the Core Unit for Bioinformatics Data Analysis of Bonn University Hospital. Non-unique peptides and single-shot proteins (proteins identified/quantified by only one peptide) were filtered-out prior to the statistical analysis. From all available fractions, only those with the least number of missing values per feature and maximum average intensity across all TMT labels were selected. The PSM-level data were then normalized and transformed to log-scale using the VSN package^55^and then aggregated to protein-level by applying the Tukey’s median polish method. The differential expression analysis was performed using the moderated t-tests from the R package limma^56^. Contrary to the normal t-test that calculates variance for each feature (here protein) independently, the moderated t-test borrows information across all proteins, which leads to more accurate and stable variance estimates, particularly when sample size is small. This reduces the impact of outliers and increases the statistical power^56^. For each statistical contrast the resulting P-values were adjusted for multiple testing and the false discovery rates (FDR) were calculated by the Benjamini–Hochberg method.

For evaluation of western blots, densitometric quantification values were determined and normalized to the respective loading control. The values were then normalized to the respective mean value of 0h starvation by log2 transformation, and a linear regression was performed with blot as a cofactor. p-values were extracted and Benjamini–Hochberg correction was applied to account for multiple testing.

The online software WEB-based GEne SeT AnaLysis Toolkit, Version 2019^57^ (https://www.webgestalt.org/) was used for overrepresentation analysis for pathways (KEGG)^20–22^ and gene ontology (cellular component) against the current (November 2023) protein-coding reference set for Mus musculus.

Statistical graphs and diagrams were created using GraphPad Prism 6.01 (https://www.graphpad.com/), FactoMineR 1.42, ggplot2 3.2.0 and Venny 2.1 (https://github.com/benfred/venn.js) software^58–61^.

### Serum preparation

After sacrifice, whole blood samples were collected and coagulation at RT was performed for 30 min. Samples were centrifuged at 18,000 × g for 10 min at 4 °C and the supernatant was transferred to a new 1.5 ml reaction tube.

### Metabolite analysis by mass spectrometry

Metabolite analysis for the determination of amino acid and acylcarnitine concentrations was performed by mass spectrometry by Dr. Eberhard & Partner medical care center (Dortmund, Germany). For acylcarnitines, 50 μl of serum samples were mixed with 200 μl of methanol extraction buffer containing stable isotope-labeled acylcarnitines, which were used as an internal standard (IS). The mixture was centrifuged at 15,000 × g for 5 min at 4 °C using a refrigerated centrifuge. The collected supernatant was concentrated and dried under a nitrogen stream at 60°C for 20 min. Following this, acylcarnitines were derivatized to their butyl esters by adding 50 μl of an anhydrous n-butanol/HCl solution to the dried samples. After the derivatization step, the samples were concentrated and dried again under the same conditions as described above. Reconstituted samples in a 100 μl acetonitrile-based solution were then ready for ESI–MS/MS measurement. 7 μl of the analyte mixture was directly injected into the MS ionization source without prior chromatographic separation. The ionization was conducted using a positive electrospray ionization (ESI +) mode (cone voltage 35 V, collision energy 25 V, capillary voltage 3000 V, source temperature 120 °C). After setting the measurement mode to parent ion scan (PIS), ions within a mass range of 200–500 Da were scanned in MS1. The ions corresponding to the mass-to-charge (m/z) ratio of butylated acylcarnitines were isolated and fragmented in the collision cell containing argon gas. The characteristic fragment ion of m/z 85 Da was selected and transferred through MS2.

For amino acids, 50 μl of serum samples were mixed with 200 μl of methanol extraction buffer. The extraction buffer contained stable isotope-labeled amino acids, which were used as an internal standard (IS). After the centrifugation at 15,000 × g for 5 min at 4 °C, 10 μl of the supernatant was mixed with 70 μl of borate buffer (pH 8.0) and 20 μl of 6-aminoquinolyl-n-hydroxysuccinimidyl carbamate solution leading to a substitution reaction at the primary and secondary amino groups. Processed samples were then ready for LC–MS/MS measurement. The serum amino acid concentrations in the processed samples were measured using an LC–MS/MS system. Briefly, the liquid chromatography (LC) separation was carried out on a C18 analytical column and the autosampler was set to 1 μl per injection. The column was equilibrated using 0.1% (v/v) formic acid. Molecules were separated at a flow rate of 0.4 ml/min during a 9 min linear gradient ranging from 1 to 95% using a solvent composed of 90% (v/v) acetonitrile and 0.1% (v/v) formic acid. The LC system was connected to a tandem mass spectrometer. The multiple reaction monitoring (MRM) mode was used for the detection of the amino acids (cone voltage 25 V, collision energy 20 V, capillary voltage 400 V, source temperature 150 °C), where only defined ions were scanned in MS1. After the fragmentation in the collision cell containing argon gas, the selected fragment ions were transferred through MS2. The internal standard (IS) was used to compensate potential effects caused by co-eluting matrix components such as the changes in the ionization and chromatographic response of target amino acids^62^. By calculating the peak area of the recorded chromatogram, the respective peaks provided quantitative information about the analyzed amino acids. The quantitative analysis was performed using a 7-point calibration standard and a blank. The calculation was based on a linear calibration function.

### Preparation of protein extracts

Mouse tissues were weighed and homogenized in five volumes of ice-cold tissue homogenization buffer (10 mM Tris–HCl pH 7.4 250 mM sucrose, 1 mM EDTA, protease and phosphatase inhibitor cocktails (Roche Diagnostics, Rotkreuz, Switzerland). After douncing for 20 strokes using a 1 ml dounce homogenizer, 1% (v/v) Triton X-100 was added to the homogenate and incubated for 1 h on ice. For a separation into soluble and insoluble Triton X-100 fractions, the mixture was centrifuged at 15.000 × g for 15 min at 4 °C. After centrifugation, the insoluble fraction (pellet) was discarded and the soluble fraction (supernatant) was transferred into a new 1.5 ml reaction tube for protein concentration determination or stored at -20 °C.

### Western blotting

Equal protein amounts of samples were heated with 1 × Laemmli^63^ for 5 min at 95 °C, separated by SDS-PAGE and blotted on either nitrocellulose (NC) or polyvinylidene fluoride (PVDF) membranes. After blocking with 5% milk powder solved in Tris-buffered saline with 0.05% Tween-20 (TBS-T) for 1 h at RT, the membranes were incubated with one of the primary antibodies: anti-4E-binding protein 1 (4E-BP1), anti-phospho-4E-BP1, anti- microtubule associated protein 1 light chain 3 beta (LC3B), anti-LAMTOR 2 and 3, anti-PRAS40, anti-NPRL2, anti-PSMB5, anti-RAGA, anti-RAGC, anti-Raptor, anti-S6-ribosomal protein (S6), anti VDAC1 (all Cell Signaling technology, Danvers, USA), anti-CPT2, anti-PSMA7, anti-WASHC4 (all Proteintech, Manchester, UK), anti-GAPDH, anti-TPP1 (Santa Cruz, Dallas, USA) overnight at 4 °C. Following a further incubation for 45 min with horseradish peroxidase-coupled secondary antibodies, blots were developed with enhanced chemiluminescence solution. Densitometric measurements of band intensity were performed with Fusion Capt advance software Version 16.15 (Vilber, Marne-la-Vallée, France) to quantify band intensities.

### Measurement of blood glucose and β-hydroxy butyrate levels

Blood glucose levels were used immediately after blood collection according to the manufacturer’s instructions with an Accu-Check guide instrument. The test results were obtained in mg/dl. β-hydroxy butyrate levels were determined in serum and the β-hydroxy butyrate assay kit (Abcam, Cambride, UK) was used according to the manufacturer’s instructions.

### RNA isolation and real-time PCR analysis

All RNA isolation steps were performed on ice and mice liver tissues were placed on dry ice, unless stated differently. Tissue samples were resuspended in 500 μl TRIzol reagent^64^ and homogenized at low speed using Ultra Thurrax T 10 basic. After 5 min incubation at RT, 100 μl chloroform was added and the samples were vortexed for 15 s. Samples were again incubated under the same conditions as described above and then centrifuged for 15 min at 16,000 × g at 4 °C, creating three phases within each sample. The upper mRNA phase was carefully transferred to a new 1.5 ml reaction tube and the remainder of the sample was discarded. After adding 500 μl of isopropanol, samples were incubated for 15 min at RT and centrifuged again for 15 min at 16,000 × g at 4 °C. The resulting pellet was washed with 700 μl of 70% ethanol. Remaining ethanol was removed, pellets were dried at RT and resuspended in 40 μl nuclease-free water. Samples were stored at -80 °C and the concentration and purity of isolated RNA were measured using a spectrophotometer. To perform a cDNA synthesis, RevertAid™ H Minus First Strand cDNA Synthesis Kit was carried out as described by the manufacturer’s instructions. To amplify the gene of interest (GOI), appropriate forward and reverse primers were selected and a primer test PCR was performed. For quantitative real-time PCR, SYBR© Select Master Mix was used to detect the amplified gene by increased fluorescence intensities using the SYBR GreenER™ dye. As recommended by the manufacturer, 125 ng cDNA was pipetted in triplicates into a 96-well microplate together with the reaction mixture. The reaction was conducted by the 7300 Real-Time PCR System using a thermocycling program (50°C 2’, 95°C 2’, 40x(95°C 15’’, 60°C 1’)). Data analysis was performed by 7300 System SDS RQ Study 1.4 software, and the 2 − ΔΔCt method^65^ was used to analyze the changes and relative gene expression. Together with each target gene reaction, an internal control such as actin as well as water control reactions were performed in triplicates.

## Supplementary Information.

Supplementary Information 1.

Supplementary Information 2.

Supplementary Information 3.

## Data Availability

Mass spectrometric data have been uploaded to the public repository https://www.proteomexchange.org/ with the dataset identifier PXD047158.

## References

[1] Xu, H. & Ren, D. Lysosomal physiology. *Annu Rev Physiol* **77**, 57–80. 10.1146/annurev-physiol-021014-071649 (2015). 25668017 10.1146/annurev-physiol-021014-071649 PMC4524569

[2] De Duve, C., Pressman, B. C., Gianetto, R., Wattiaux, R. & Appelmans, F. Tissue fractionation studies. 6. Intracellular distribution patterns of enzymes in rat-liver tissue. *Biochem J* **60**, 604–617. 10.1042/bj0600604 (1955). 13249955 10.1042/bj0600604 PMC1216159

[3] Ballabio, A. & Bonifacino, J. S. Lysosomes as dynamic regulators of cell and organismal homeostasis. *Nat Rev Mol Cell Biol* **21**, 101–118. 10.1038/s41580-019-0185-4 (2020). 31768005 10.1038/s41580-019-0185-4

[4] Liu, G. Y. & Sabatini, D. M. mTOR at the nexus of nutrition, growth, ageing and disease. *Nat Rev Mol Cell Biol* **21**, 183–203. 10.1038/s41580-019-0199-y (2020). 31937935 10.1038/s41580-019-0199-y PMC7102936

[5] Sengupta, S., Peterson, T. R. & Sabatini, D. M. Regulation of the mTOR complex 1 pathway by nutrients, growth factors, and stress. *Mol Cell* **40**, 310–322. 10.1016/j.molcel.2010.09.026 (2010). 20965424 10.1016/j.molcel.2010.09.026 PMC2993060

[6] Saxton, R. A. & Sabatini, D. M. mTOR Signaling in Growth, Metabolism, and Disease. *Cell* **168**, 960–976. 10.1016/j.cell.2017.02.004 (2017). 28283069 10.1016/j.cell.2017.02.004 PMC5394987

[7] Saito, K., Araki, Y., Kontani, K., Nishina, H. & Katada, T. Novel role of the small GTPase Rheb: its implication in endocytic pathway independent of the activation of mammalian target of rapamycin. *J Biochem* **137**, 423–430. 10.1093/jb/mvi046 (2005). 15809346 10.1093/jb/mvi046

[8] Wang, S. et al. Metabolism Lysosomal amino acid transporter SLC38A9 signals arginine sufficiency to mTORC1. *Science* **347**, 188–194. 10.1126/science.1257132 (2015). 25567906 10.1126/science.1257132 PMC4295826

[9] Kim, J. & Guan, K. L. mTOR as a central hub of nutrient signalling and cell growth. *Nat Cell Biol* **21**, 63–71. 10.1038/s41556-018-0205-1 (2019). 30602761 10.1038/s41556-018-0205-1

[10] Napolitano, G., Di Malta, C. & Ballabio, A. Non-canonical mTORC1 signaling at the lysosome. *Trends Cell Biol* **32**, 920–931. 10.1016/j.tcb.2022.04.012 (2022). 35654731 10.1016/j.tcb.2022.04.012

[11] Sancak, Y. et al. Ragulator-Rag complex targets mTORC1 to the lysosomal surface and is necessary for its activation by amino acids. *Cell* **141**, 290–303. 10.1016/j.cell.2010.02.024 (2010). 20381137 10.1016/j.cell.2010.02.024 PMC3024592

[12] Tan, H. W. S., Sim, A. Y. L. & Long, Y. C. Glutamine metabolism regulates autophagy-dependent mTORC1 reactivation during amino acid starvation. *Nat Commun* **8**, 338. 10.1038/s41467-017-00369-y (2017). 28835610 10.1038/s41467-017-00369-y PMC5569045

[13] Mizushima, N., Yamamoto, A., Matsui, M., Yoshimori, T. & Ohsumi, Y. In vivo analysis of autophagy in response to nutrient starvation using transgenic mice expressing a fluorescent autophagosome marker. *Mol Biol Cell* **15**, 1101–1111. 10.1091/mbc.e03-09-0704 (2004). 14699058 10.1091/mbc.E03-09-0704 PMC363084

[14] Song, S. et al. Peroxisome proliferator activated receptor alpha (PPARalpha) and PPAR gamma coactivator (PGC-1alpha) induce carnitine palmitoyltransferase IA (CPT-1A) via independent gene elements. *Mol Cell Endocrinol* **325**, 54–63. 10.1016/j.mce.2010.05.019 (2010). 20638986 10.1016/j.mce.2010.05.019 PMC3160239

[15] Newman, J. C. & Verdin, E. beta-Hydroxybutyrate: A Signaling Metabolite. *Annu Rev Nutr* **37**, 51–76. 10.1146/annurev-nutr-071816-064916 (2017). 28826372 10.1146/annurev-nutr-071816-064916 PMC6640868

[16] Nagasawa, T., Yoshizawa, F. & Nishizawa, N. Plasma N tau-methylhistidine concentration is a sensitive index of myofibrillar protein degradation during starvation in rats. *Biosci Biotechnol Biochem* **60**, 501–502. 10.1271/bbb.60.501 (1996). 8901113 10.1271/bbb.60.501

[17] Leighton, F. et al. The large-scale separation of peroxisomes, mitochondria, and lysosomes from the livers of rats injected with triton WR-1339. Improved isolation procedures, automated analysis, biochemical and morphological properties of fractions. *J Cell Biol* **37**, 482–513. 10.1083/jcb.37.2.482 (1968). 4297786 10.1083/jcb.37.2.482 PMC2107417

[18] Ohsumi, Y., Ishikawa, T. & Kato, K. A rapid and simplified method for the preparation of lysosomal membranes from rat liver. *J Biochem* **93**, 547–556 (1983). 6841352

[19] Trouet, A. Isolation of modified liver lysosomes. *Methods Enzymol* **31**, 323–329. 10.1016/0076-6879(74)31034-8 (1974). 4370710 10.1016/0076-6879(74)31034-8

[20] Kanehisa, M. Toward understanding the origin and evolution of cellular organisms. *Protein Sci* **28**, 1947–1951. 10.1002/pro.3715 (2019). 31441146 10.1002/pro.3715 PMC6798127

[21] Kanehisa, M., Furumichi, M., Sato, Y., Kawashima, M. & Ishiguro-Watanabe, M. KEGG for taxonomy-based analysis of pathways and genomes. *Nucleic Acids Res* **51**, D587–D592. 10.1093/nar/gkac963 (2023). 36300620 10.1093/nar/gkac963 PMC9825424

[22] Kanehisa, M. & Goto, S. KEGG: kyoto encyclopedia of genes and genomes. *Nucleic Acids Res* **28**, 27–30. 10.1093/nar/28.1.27 (2000). 10592173 10.1093/nar/28.1.27 PMC102409

[23] Wyant, G. A. et al. NUFIP1 is a ribosome receptor for starvation-induced ribophagy. *Science* **360**, 751–758. 10.1126/science.aar2663 (2018). 29700228 10.1126/science.aar2663 PMC6020066

[24] Kim, J., Kundu, M., Viollet, B. & Guan, K. L. AMPK and mTOR regulate autophagy through direct phosphorylation of Ulk1. *Nat Cell Biol* **13**, 132–141. 10.1038/ncb2152 (2011). 21258367 10.1038/ncb2152 PMC3987946

[25] Vargas, J. N. S., Hamasaki, M., Kawabata, T., Youle, R. J. & Yoshimori, T. The mechanisms and roles of selective autophagy in mammals. *Nat Rev Mol Cell Biol* **24**, 167–185. 10.1038/s41580-022-00542-2 (2023). 36302887 10.1038/s41580-022-00542-2

[26] Jensen, T. L., Kiersgaard, M. K., Sorensen, D. B. & Mikkelsen, L. F. Fasting of mice: a review. *Lab Anim* **47**, 225–240. 10.1177/0023677213501659 (2013). 24025567 10.1177/0023677213501659

[27] Ezaki, J. et al. Liver autophagy contributes to the maintenance of blood glucose and amino acid levels. *Autophagy* **7**, 727–736. 10.4161/auto.7.7.15371 (2011). 21471734 10.4161/auto.7.7.15371 PMC3149698

[28] Mortimore, G. E., Hutson, N. J. & Surmacz, C. A. Quantitative correlation between proteolysis and macro- and microautophagy in mouse hepatocytes during starvation and refeeding. *Proc Natl Acad Sci U S A* **80**, 2179–2183. 10.1073/pnas.80.8.2179 (1983). 6340116 10.1073/pnas.80.8.2179 PMC393781

[29] Kristensen, A. R. et al. Ordered organelle degradation during starvation-induced autophagy. *Mol Cell Proteomics* **7**, 2419–2428. 10.1074/mcp.M800184-MCP200 (2008). 18687634 10.1074/mcp.M800184-MCP200

[30] Cuervo, A. M., Palmer, A., Rivett, A. J. & Knecht, E. Degradation of proteasomes by lysosomes in rat liver. *Eur J Biochem* **227**, 792–800. 10.1111/j.1432-1033.1995.tb20203.x (1995). 7867640 10.1111/j.1432-1033.1995.tb20203.x

[31] Cohen-Kaplan, V. et al. p62- and ubiquitin-dependent stress-induced autophagy of the mammalian 26S proteasome. *Proc Natl Acad Sci U S A* **113**, E7490–E7499. 10.1073/pnas.1615455113 (2016). 27791183 10.1073/pnas.1615455113 PMC5127335

[32] Gomes, L. C., Di Benedetto, G. & Scorrano, L. During autophagy mitochondria elongate, are spared from degradation and sustain cell viability. *Nat Cell Biol* **13**, 589–598. 10.1038/ncb2220 (2011). 21478857 10.1038/ncb2220 PMC3088644

[33] Sengupta, S., Peterson, T. R., Laplante, M., Oh, S. & Sabatini, D. M. mTORC1 controls fasting-induced ketogenesis and its modulation by ageing. *Nature* **468**, 1100–1104 (2010). https://doi.org:10.1038/nature09584 10.1038/nature09584 21179166

[34] Groenewoud, M. J. & Zwartkruis, F. J. Rheb and Rags come together at the lysosome to activate mTORC1. *Biochem Soc Trans* **41**, 951–955 (2013). https://doi.org:10.1042/BST20130037 10.1042/BST20130037 23863162

[35] Tsujimoto, K., Takamatsu, H. & Kumanogoh, A. The Ragulator complex: delving its multifunctional impact on metabolism and beyond. *Inflamm Regen* **43**, 28 (2023). https://doi.org:10.1186/s41232-023-00278-2 10.1186/s41232-023-00278-2 PMC10175929 37173755

[36] Nowosad, A. *et al.* p27 controls Ragulator and mTOR activity in amino acid-deprived cells to regulate the autophagy-lysosomal pathway and coordinate cell cycle and cell growth. *Nat Cell Biol* **22**, 1076–1090 (2020). https://doi.org:10.1038/s41556-020-0554-4 10.1038/s41556-020-0554-4 32807902

[37] Yu, L. *et al.* Termination of autophagy and reformation of lysosomes regulated by mTOR. *Nature* **465**, 942–946 (2010). https://doi.org:10.1038/nature09076 10.1038/nature09076 PMC2920749 20526321

[38] Buel, G. R., Dang, H. Q., Asara, J. M., Blenis, J. & Mutvei, A. P. Prolonged deprivation of arginine or leucine induces PI3K/Akt-dependent reactivation of mTORC1. *J Biol Chem* **298**, 102030 (2022). https://doi.org:10.1016/j.jbc.2022.102030 10.1016/j.jbc.2022.102030 PMC9194872 35577075

[39] Murata, Y., Uehara, Y. & Hosoi, Y. Activation of mTORC1 under nutrient starvation conditions increases cellular radiosensitivity in human liver cancer cell lines, HepG2 and HuH6. *Biochem Biophys Res Commun* **468**, 684–690 (2015). https://doi.org:10.1016/j.bbrc.2015.11.016 10.1016/j.bbrc.2015.11.016 26585486

[40] Risson, V. *et al.* Muscle inactivation of mTOR causes metabolic and dystrophin defects leading to severe myopathy. *J Cell Biol* **187**, 859–874 (2009). https://doi.org:10.1083/jcb.200903131 10.1083/jcb.200903131 PMC2806319 20008564

[41] Bentzinger, C. F. *et al.* Skeletal muscle-specific ablation of raptor, but not of rictor, causes metabolic changes and results in muscle dystrophy. *Cell Metab* **8**, 411–424 (2008). https://doi.org:10.1016/j.cmet.2008.10.002 10.1016/j.cmet.2008.10.002 19046572

[42] Watford, M. (2015). Starvation: Metabolic Changes. *eLS.*10.1002/9780470015902.a0000642.pub2

[43] Simpson, I. A. *et al.* The facilitative glucose transporter GLUT3: 20 years of distinction. *Am J Physiol Endocrinol Metab* **295**, E242–253 (2008). https://doi.org:10.1152/ajpendo.90388.2008 10.1152/ajpendo.90388.2008 PMC2519757 18577699

[44] Wattiaux, R., Wibo, M. & Baudhuin, P. Effect of the injection of Triton WR 1339 on the hepatic lysosomes of the rat. *Arch Int Physiol Biochim* **71**, 140–142 (1963). 13999241

[45] Tuermer, A. *et al.* CLN6 deficiency causes selective changes in the lysosomal protein composition. *Proteomics* **21**, e2100043 (2021). https://doi.org:10.1002/pmic.202100043 10.1002/pmic.202100043 34432360

[46] Markmann, S. *et al.* Quantitative Proteome Analysis of Mouse Liver Lysosomes Provides Evidence for Mannose 6-phosphate-independent Targeting Mechanisms of Acid Hydrolases in Mucolipidosis II. *Mol Cell Proteomics* **16**, 438–450 (2017). https://doi.org:10.1074/mcp.M116.063636 10.1074/mcp.M116.063636 PMC5341004 28062798

[47] Massa Lopez, D. *et al.* The lysosomal transporter MFSD1 is essential for liver homeostasis and critically depends on its accessory subunit GLMP. *Elife* **8** (2019). https://doi.org:10.7554/eLife.50025 10.7554/eLife.50025 PMC6819133 31661432

[48] Leon, I. R., Schwammle, V., Jensen, O. N. & Sprenger, R. R. Quantitative assessment of in-solution digestion efficiency identifies optimal protocols for unbiased protein analysis. *Mol Cell Proteomics* **12**, 2992–3005 (2013). https://doi.org:10.1074/mcp.M112.025585 10.1074/mcp.M112.025585 PMC3790306 23792921

[49] Masuda, T., Tomita, M. & Ishihama, Y. Phase transfer surfactant-aided trypsin digestion for membrane proteome analysis. *J Proteome Res* **7**, 731–740 (2008). https://doi.org:10.1021/pr700658q 10.1021/pr700658q 18183947

[50] Manza, L. L., Stamer, S. L., Ham, A. J., Codreanu, S. G. & Liebler, D. C. Sample preparation and digestion for proteomic analyses using spin filters. *Proteomics* **5**, 1742–1745 (2005). https://doi.org:10.1002/pmic.200401063 10.1002/pmic.200401063 15761957

[51] Wisniewski, J. R., Zougman, A., Nagaraj, N. & Mann, M. Universal sample preparation method for proteome analysis. *Nat Methods* **6**, 359–362 (2009). https://doi.org:10.1038/nmeth.1322 10.1038/nmeth.1322 19377485

[52] Rappsilber, J., Mann, M. & Ishihama, Y. Protocol for micro-purification, enrichment, pre-fractionation and storage of peptides for proteomics using StageTips. *Nat Protoc* **2**, 1896–1906 (2007). https://doi.org:10.1038/nprot.2007.261 10.1038/nprot.2007.261 17703201

[53] Kall, L., Storey, J. D., MacCoss, M. J. & Noble, W. S. Assigning significance to peptides identified by tandem mass spectrometry using decoy databases. *J Proteome Res* **7**, 29–34 (2008). https://doi.org:10.1021/pr700600n 10.1021/pr700600n 18067246

[54] R Core Team. R: A Language and Environment for Statistical Computing. *R Foundation for Statistical Computing* (2018).

[55] Huber, W., von Heydebreck, A., Sultmann, H., Poustka, A. & Vingron, M. Variance stabilization applied to microarray data calibration and to the quantification of differential expression. *Bioinformatics* **18 Suppl 1**, S96–104 (2002). https://doi.org:10.1093/bioinformatics/18.suppl_1.s96 10.1093/bioinformatics/18.suppl_1.s96 12169536

[56] Ritchie, M. E. *et al.* limma powers differential expression analyses for RNA-sequencing and microarray studies. *Nucleic Acids Res* **43**, e47 (2015). https://doi.org:10.1093/nar/gkv007 10.1093/nar/gkv007 PMC4402510 25605792

[57] Liao, Y., Wang, J., Jaehnig, E. J., Shi, Z. & Zhang, B. WebGestalt 2019: gene set analysis toolkit with revamped UIs and APIs. *Nucleic Acids Res* **47**, W199-W205 (2019). https://doi.org:10.1093/nar/gkz401 10.1093/nar/gkz401 PMC6602449 31114916

[58] Olivero, J. C. Venny. An interactive tool for comparing lists with Venn’s diagrams. (2007–2015).

[59] Lê, S. FactoMineR: AN R Package for Multivariate Analysis. *Journal of Statistical Software* **25**, 1–18 (2008). https://doi.org:10.18637/jss.v025.i01

[60] Gu, Z., Eils, R. & Schlesner, M. Complex heatmaps reveal patterns and correlations in multidimensional genomic data. *Bioinformatics* **32**, 2847–2849 (2016). https://doi.org:10.1093/bioinformatics/btw313 10.1093/bioinformatics/btw313 27207943

[61] Wickham, H. *ggplot2*. (Springer-book, 2016).

[62] Panuwet, P. *et al.* Biological Matrix Effects in Quantitative Tandem Mass Spectrometry-Based Analytical Methods: Advancing Biomonitoring. *Crit Rev Anal Chem* **46**, 93–105 (2016). https://doi.org:10.1080/10408347.2014.980775 10.1080/10408347.2014.980775 PMC4695332 25562585

[63] Laemmli, U. K. Cleavage of structural proteins during the assembly of the head of bacteriophage T4. *Nature* **227**, 680–685 (1970). 5432063 10.1038/227680a0

[64] Chomczynski, P. & Sacchi, N. Single-step method of RNA isolation by acid guanidinium thiocyanate-phenol-chloroform extraction. *Anal Biochem* **162**, 156–159 (1987). https://doi.org:10.1006/abio.1987.9999 10.1006/abio.1987.9999 2440339

[65] Livak, K. J. & Schmittgen, T. D. Analysis of relative gene expression data using real-time quantitative PCR and the 2(-Delta Delta C(T)) Method. *Methods* **25**, 402–408 (2001). https://doi.org:10.1006/meth.2001.1262 10.1006/meth.2001.1262 11846609

