# Publisher Correction: Starvation-induced metabolic rewiring affects mTORC1 composition in vivo

**DOI:** 10.1038/s41598-025-87904-w

**Published:** 2025-01-29

**Authors:** Edgar Kaade, Simone Mausbach, Nina Erps, Marc Sylvester, Farhad Shakeri, Ron D. Jachimowicz, Volkmar Gieselmann, Melanie Thelen

**Affiliations:** 1 Institute for Biochemistry and Molecular Biology, Medical Faculty, Rheinische Friedrich-Wilhelms-University of Bonn, 53115 Bonn, Germany; 2 Max-Planck Institute for Biology of Ageing, Joseph Stelzmann Str. 9B, 50931 Cologne, Germany; 3 Core Facility Analytical Proteomics, Medical Faculty , Rheinische Friedrich-Wilhelms-University of Bonn, 53115 Bonn, Germany; 4 Institute for Medical Biometry, Informatics and Epidemiology, Medical Faculty, Rheinische Friedrich-Wilhelms-University of Bonn, Venusberg-Campus 1, 53127 Bonn, Germany; 5 Institute for Genomic Statistics and Bioinformatics, Medical Faculty, Rheinische Friedrich-Wilhelms-University of Bonn, Venusberg-Campus 1, 53127 Bonn, Germany; 6 Department I of Internal Medicine, Center for Integrated Oncology Aachen Bonn Cologne Duesseldorf (CIO ABCD), University of Cologne, Cologne, Germany; 7 Cologne Excellence Cluster on Cellular Stress Response in Aging-Associated Diseases, University of Cologne, Cologne, Germany

Correction to: *Scientific Reports* 10.1038/s41598-024-78873-7, published online 16 November 2024

The original version of this Article contained an error in which the surnames and given names of the authors were reversed. The correct names are Edgar Kaade, Simone Mausbach, Nina Erps, Marc Sylvester, Farhad Shakeri, Volkmar Gieselmann and Melanie Thelen.

The original Article has been corrected.

