# Supplementary material for: Starvation-induced metabolic rewiring affects mTORC1 composition in vivo
Source: Sci Rep. 2024 Nov 16;14:28296. doi: 10.1038/s41598-024-78873-7 (PMC11569187; doi:10.1038/s41598-024-78873-7)
Supplement: Supplementary file 1 — Supplementary Information 1. [file 41598_2024_78873_MOESM1_ESM.pdf]

Starvation-induced metabolic rewiring affects mTORC1 composition *in vivo*

Kaade, Edgar[1]; Mausbach, Simone[1]; Erps, Nina[2]; Sylvester, Marc[1,3]; Shakeri, Farhad[4,5]; Jachimowicz, Ron D.[2,6,7]; Gieselmann, Volkmar[1]; Thelen, Melanie[1,2,6]*

[1] Institute for Biochemistry and Molecular Biology, Medical Faculty, Rheinische Friedrich-Wilhelms-University of Bonn, 53115 Bonn, Germany
[2] Max-Planck Institute for Biology of Ageing, 50931 Cologne, Germany
[3] Core Facility Analytical Proteomics, Medical Faculty, Rheinische Friedrich-Wilhelms-University of Bonn, 53115 Bonn,Germany
[4] Institute for Medical Biometry, Informatics and Epidemiology, Medical Faculty, University of Bonn, Venusberg-Campus 1, 53127 Bonn, Germany
[5] Institute for Genomic Statistics and Bioinformatics, Medical Faculty, University of Bonn, Venusberg-Campus 1, 53127 Bonn, Germany
[6] Department I of Internal Medicine, Center for Integrated Oncology Aachen Bonn Cologne Duesseldorf (CIO ABCD), University of Cologne, Cologne, Germany
[7] Cologne Excellence Cluster on Cellular Stress Response in Aging-Associated Diseases, University of Cologne, Cologne, Germany

Supplementary Figures

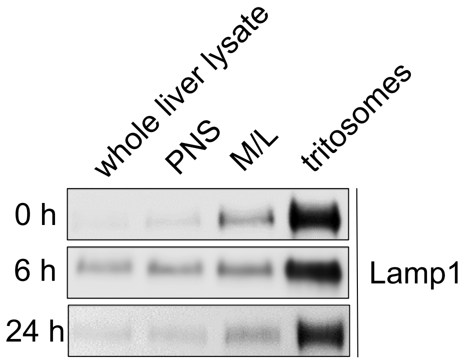

Supplementary Figure 1: Verification of comparable lysosomal enrichment after tritosome isolation by LAMP1 immunoblotting of different fractions. Equal protein amounts of each fraction were loaded. PNS= postnuclear supernatant, M/L=mitochondria/lysosome fraction.

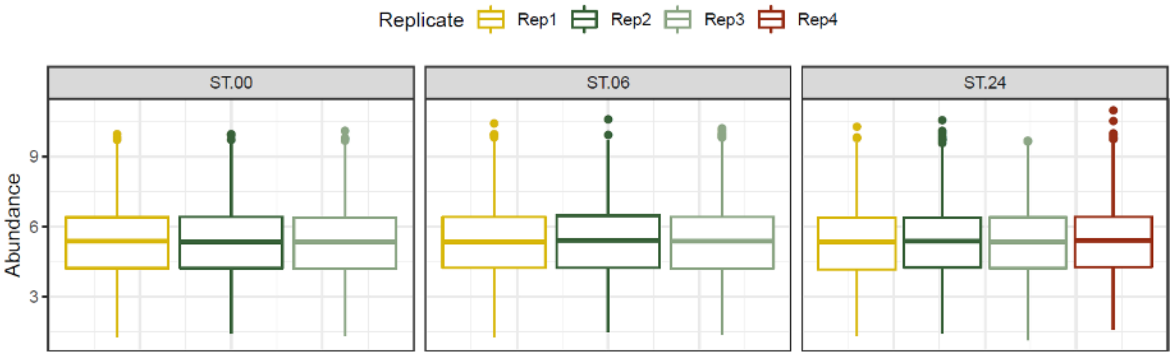

Supplementary Figure 2: Distribution of replicates after normalization. ST=Starvation. Proteomic data were normalized by the variance stabilizing normalization method (VSN). Normalized values were used to display boxplot by ggplot2 3.2.0 software. Boxplots represent every replicate of control (n=3), 6 (n=3) and 24 (n=24) hours starved mice. Summarized proteins of each replicate were represented by abundance values. Outliers are displayed as additional dots at the appropriate replicates.

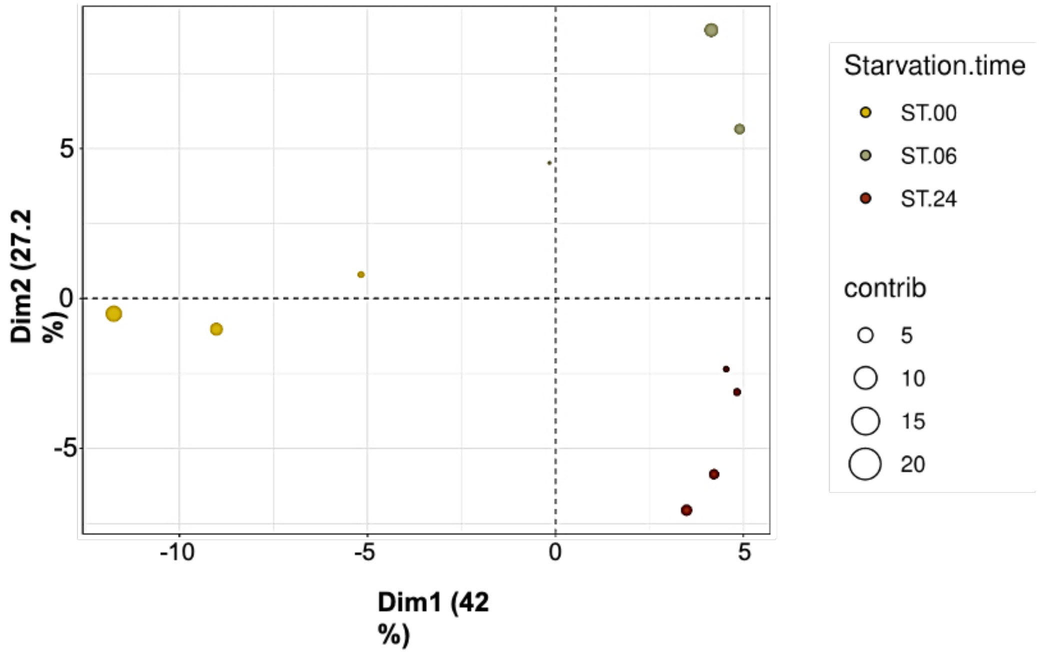

Supplementary Figure 3: Clustering of replicates visualized by boxplot. Principal component analysis plot using FactoMineR 1.42 software. Three groups of replicates demonstrating each condition are represented with a particular colored dot (Yellow: control group, green: 6 hours starved group, red: 24 hours starved group). Dot sizes diverse based on their contribution to each dimension (Dim1 and Dim2).
