# Supplementary material for: Starvation-induced metabolic rewiring affects mTORC1 composition in vivo
Source: Sci Rep. 2024 Nov 16;14:28296. doi: 10.1038/s41598-024-78873-7 (PMC11569187; doi:10.1038/s41598-024-78873-7)
Supplement: Supplementary file 3 — Supplementary Information 3. [file 41598_2024_78873_MOESM3_ESM.pdf]

Starvation-induced metabolic rewiring affects mTORC1 composition *in vivo*

Kaade, Edgar[1]; Mausbach, Simone[1]; Erps, Nina[2]; Sylvester, Marc[1,3]; Shakeri, Farhad[4,5]; Jachimowicz, Ron D. [2,6,7]; Gieselmann, Volkmar[1]; Thelen, Melanie[1,2,6]*

[1] Institute for Biochemistry and Molecular Biology, Medical Faculty, Rheinische Friedrich-Wilhelms-University of Bonn, 53115 Bonn, Germany
[2] Max-Planck Institute for Biology of Ageing, 50931 Cologne, Germany
[3] Core Facility Analytical Proteomics, Medical Faculty, Rheinische Friedrich-Wilhelms-University of Bonn, 53115 Bonn,Germany
[4] Institute for Medical Biometry, Informatics and Epidemiology, Medical Faculty, University of Bonn, Venusberg-Campus 1, 53127 Bonn, Germany
[5] Institute for Genomic Statistics and Bioinformatics, Medical Faculty, University of Bonn, Venusberg-Campus 1, 53127 Bonn, Germany
[6] Department I of Internal Medicine, Center for Integrated Oncology Aachen Bonn Cologne Duesseldorf (CIO ABCD), University of Cologne, Cologne, Germany
[7] Cologne Excellence Cluster on Cellular Stress Response in Aging-Associated Diseases, University of Cologne, Cologne, Germany

Supplementary Information – Western Blot full data

All Western Blot were developed with Fusion Chemiluminsecence developer (Vilber, France). Shown are mostly merged pictures of blot and size marker picture. Size of nearest marker band is indicated.

Figure 3

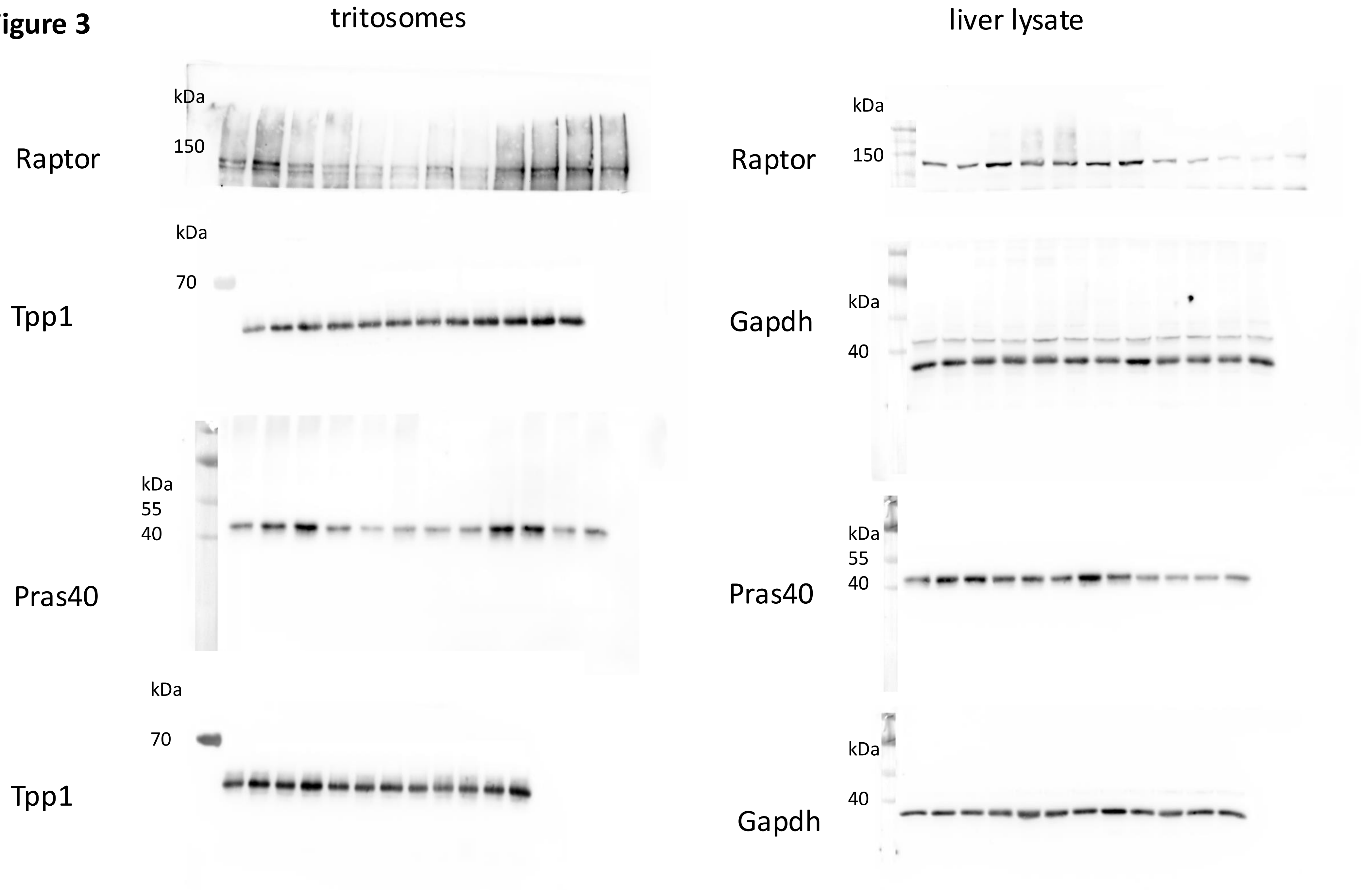
tritosomes
liver lysate
kDa
150
Raptor
kDa
70
Tpp1
kDa
55
40
Pras40
kDa
70
Tpp1
kDa
150
Raptor
kDa
40
Gapdh
kDa
55
40
Pras40
kDa
40
Gapdh

Figure 3

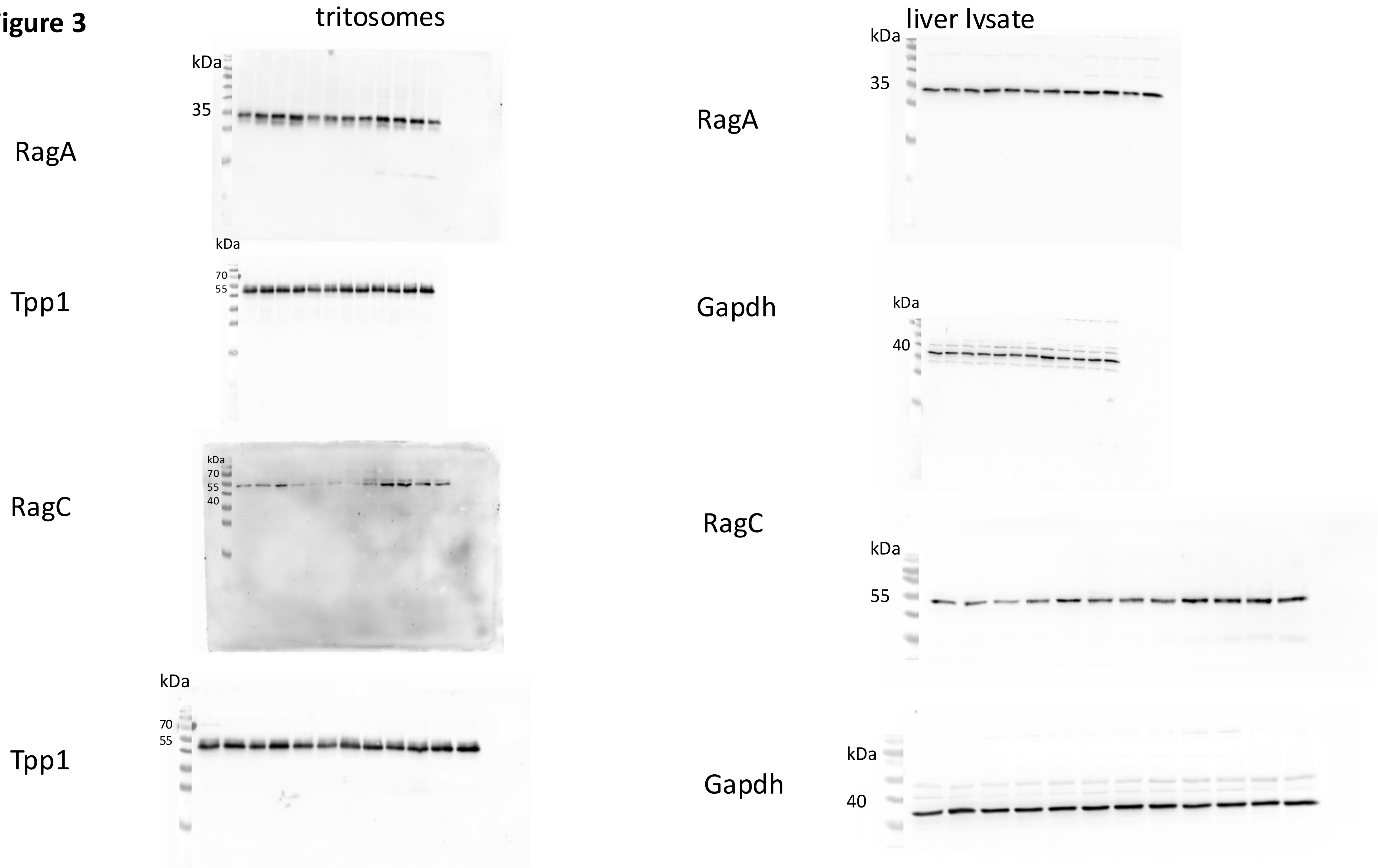

tritosomes
liver lysate
RagA
Tpp1
RagC
Tpp1
RagA
Gapdh
RagC
Gapdh
kDa
35
70
55
40

Figure 3

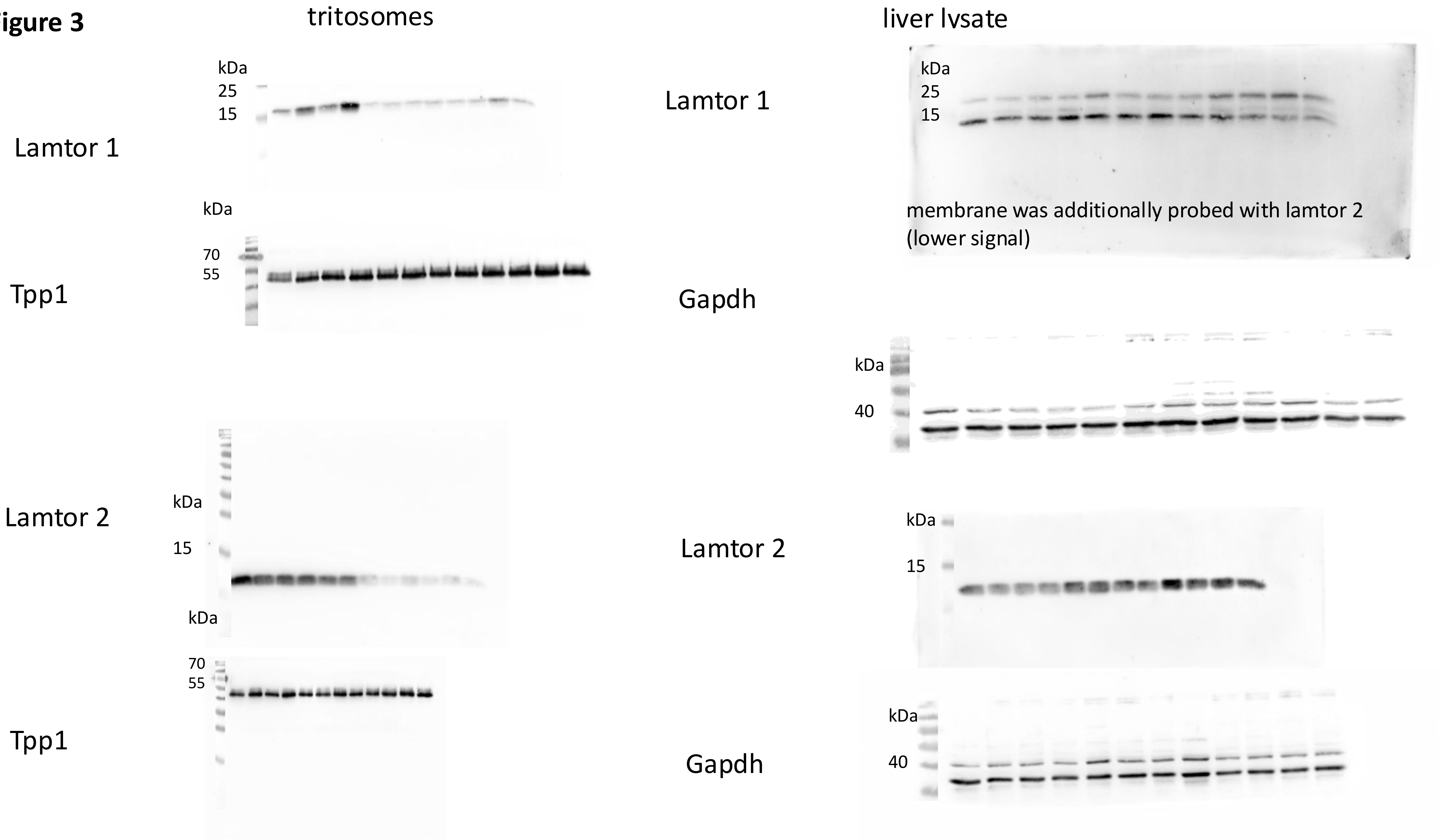
tritosomes
liver lysate
Lamtor 1
kDa
25
15
Tpp1
70
55
Lamtor 2
Gapdh
40
membrane was additionally probed with lamtor 2 (lower signal)

Figure 3

tritosomes

Lamtor 5

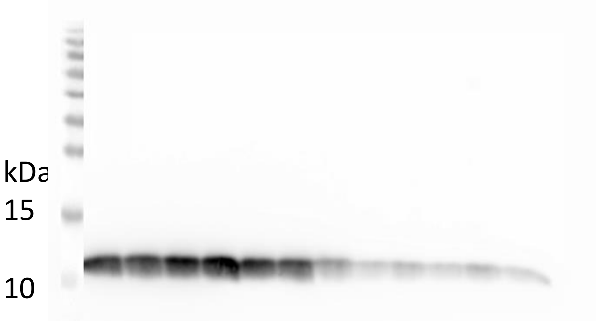

Tpp1

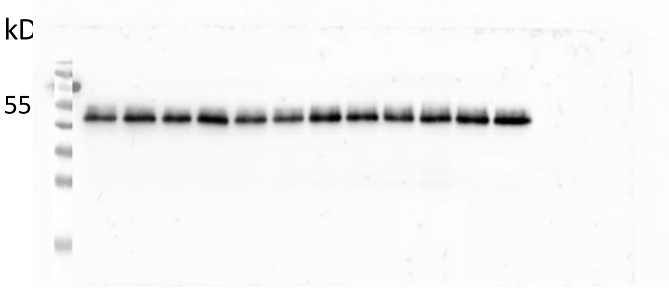

liver lysate

Lamtor 5

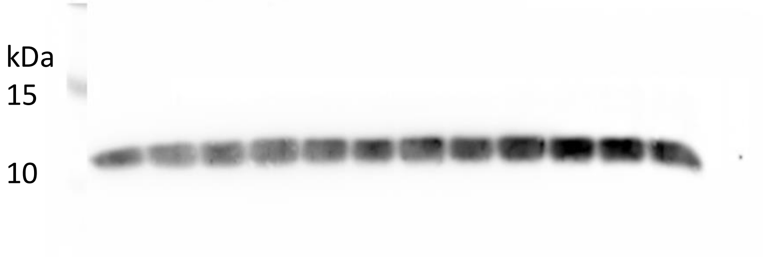

Gapdh

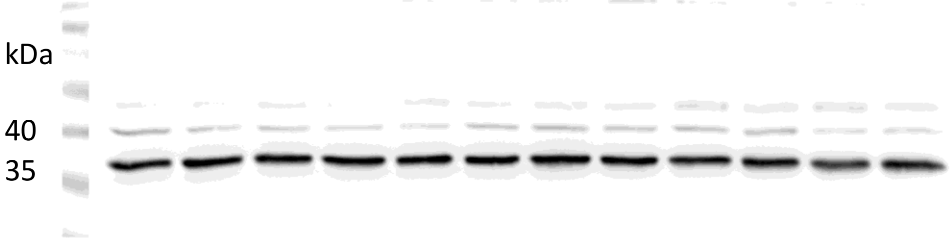

Figure 4 Panel B

Gapdh 0, 6, 9h

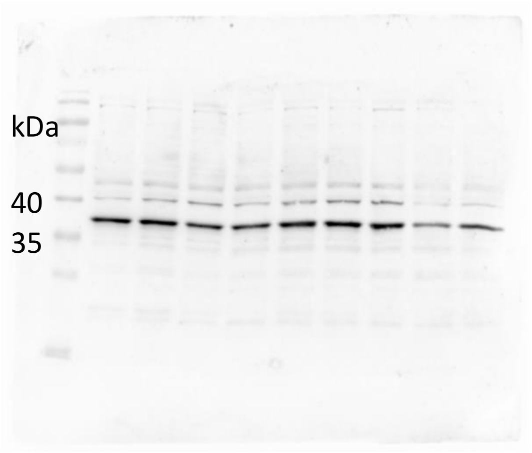

P-4E-BP1 0, 6, 9h

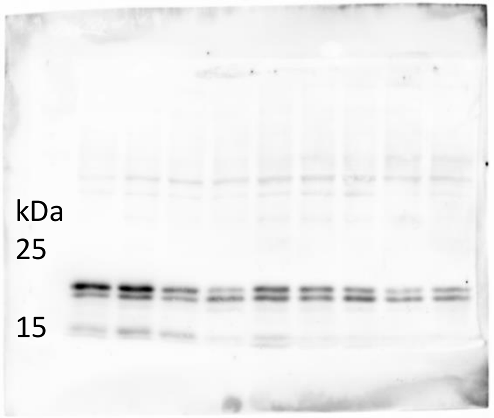

P-4E-BP1 0,12,16,20h

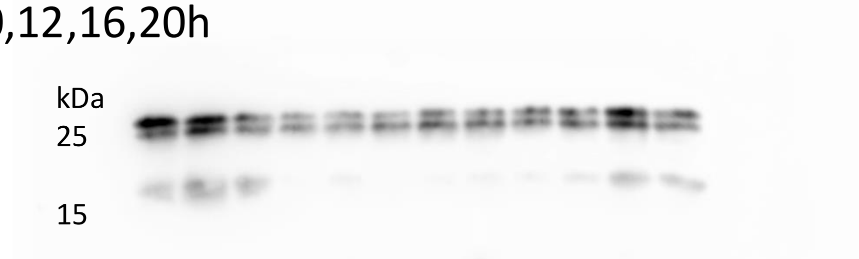

Gapdh 0,12,16,20h

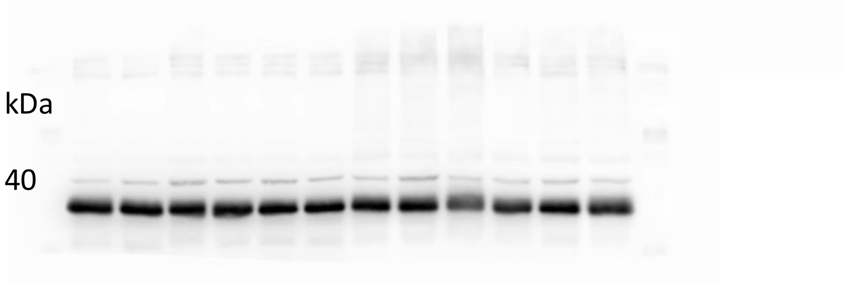

Figure 4 Panel B

4E-BP1 0,6,9 h

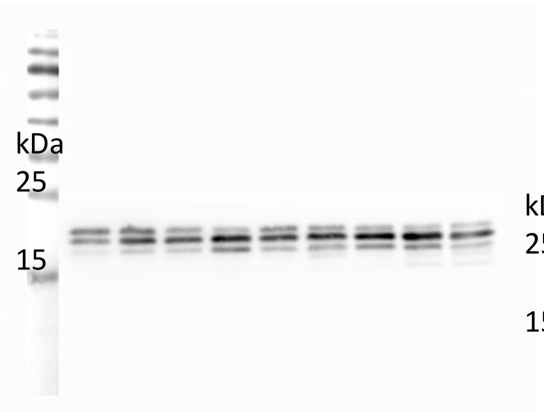

4E-BP1 0,12 h

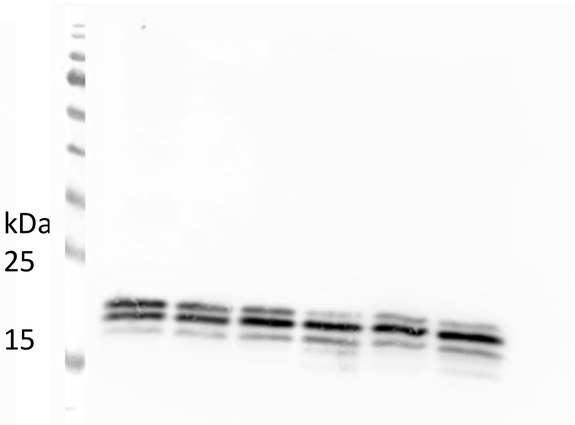

4E-BP1 0,16 h

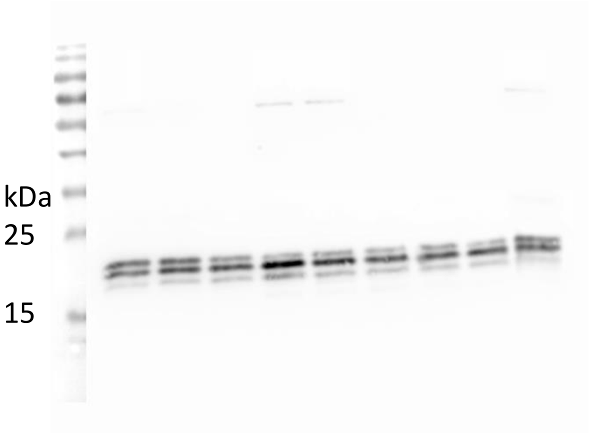

4E-BP1 0,20 h

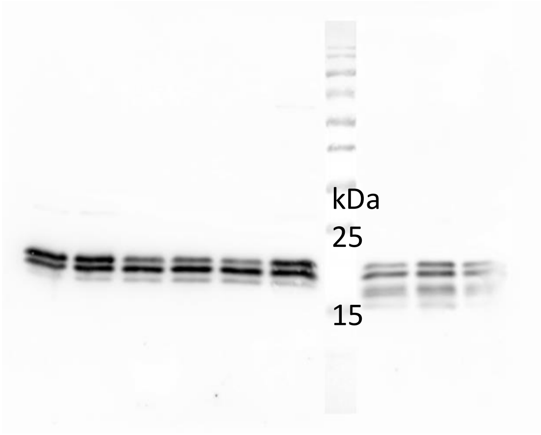

Gapdh 0, 6, 9, 12 h

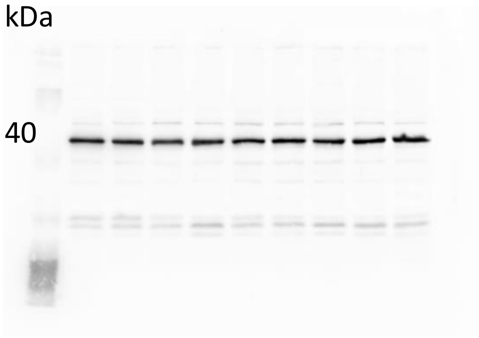

Gapdh 0,16,20 h

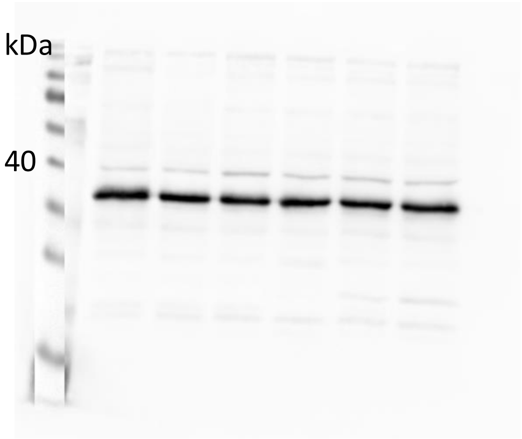

Gapdh 0,16 h

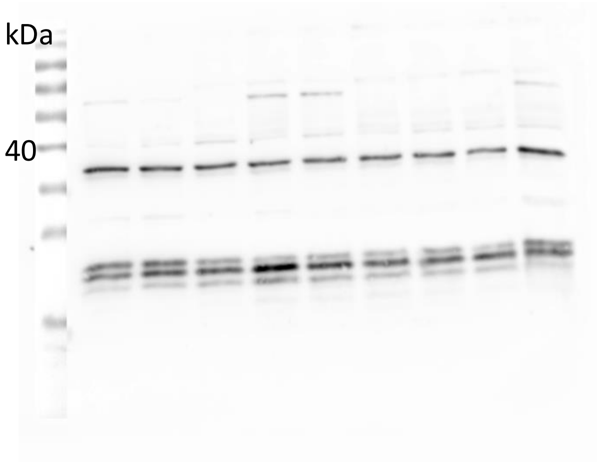

Last three lanes of blot are additional samples not displayed in this publication

Gapdh 0, 20h

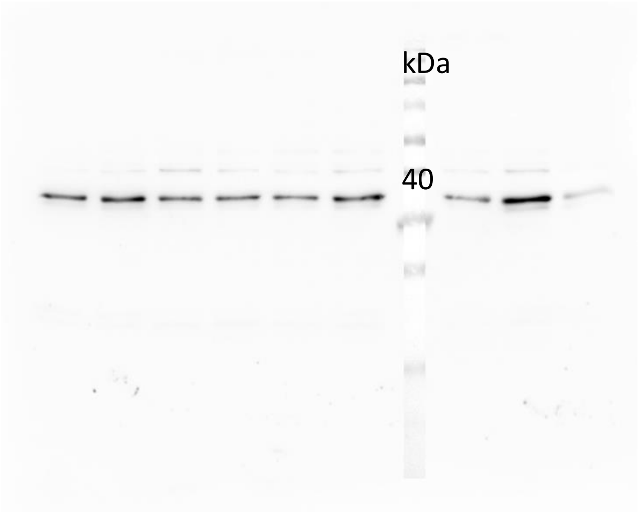

Last three lanes of blot are additional samples not displayed in this publication

Figure 4 Panel B

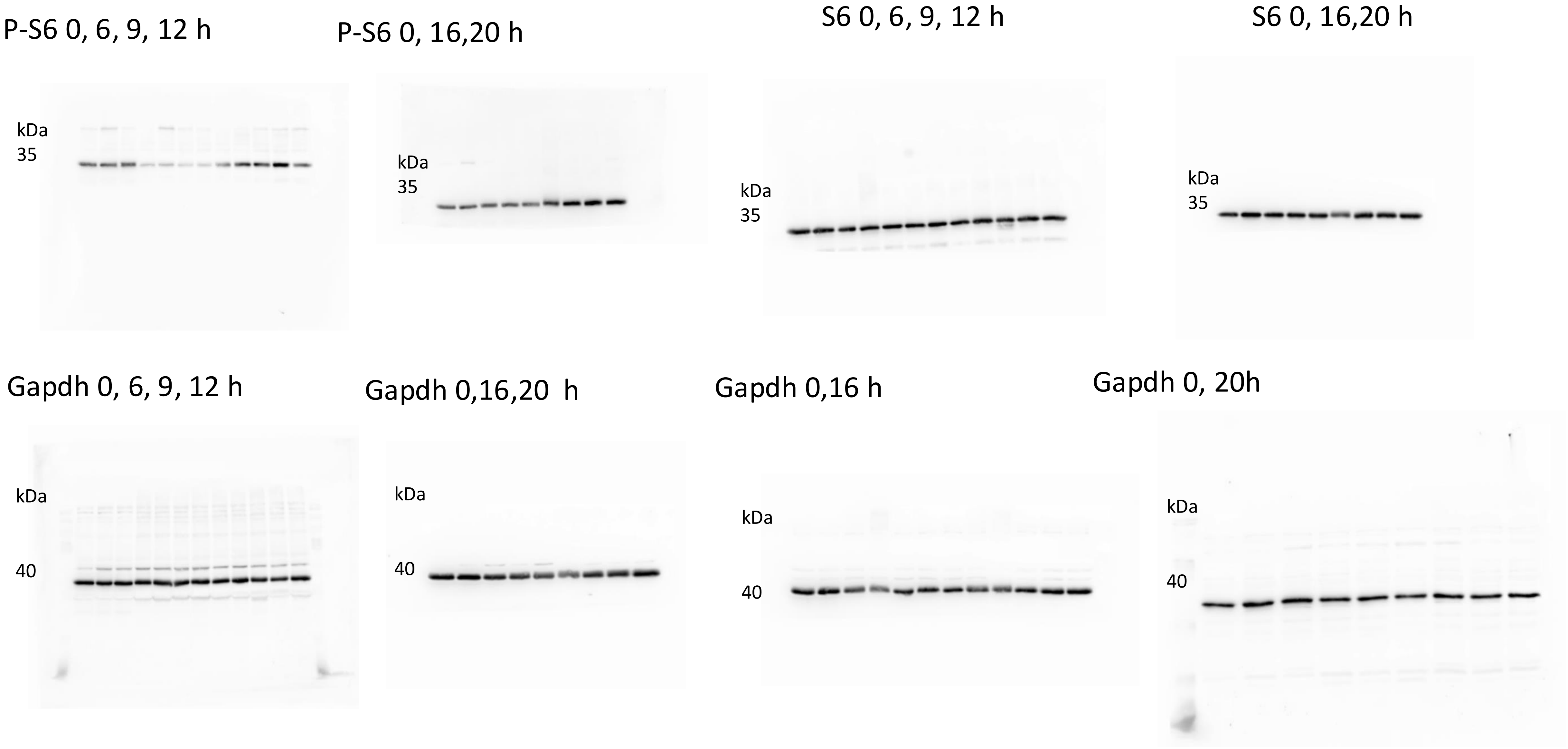

Figure 4 Panel B

Lc3-B 0, 6, 9, 12 h

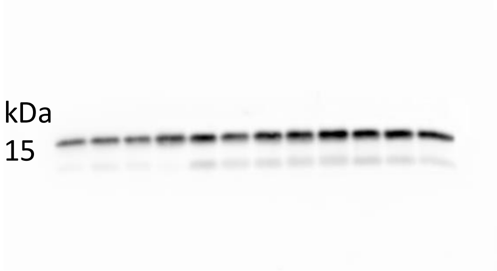

Lc3-B 0, 16,20 h

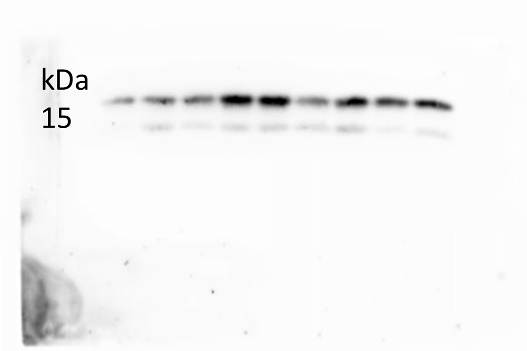

Gapdh 0, 6, 9, 12 h

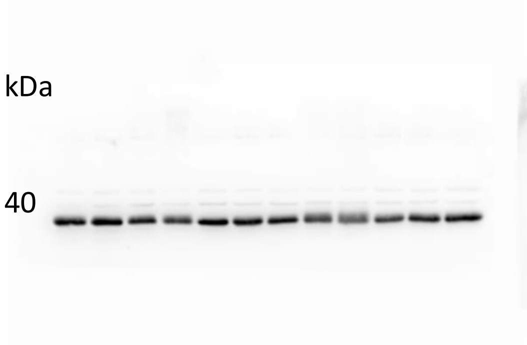

Gapdh 0,16,20 h

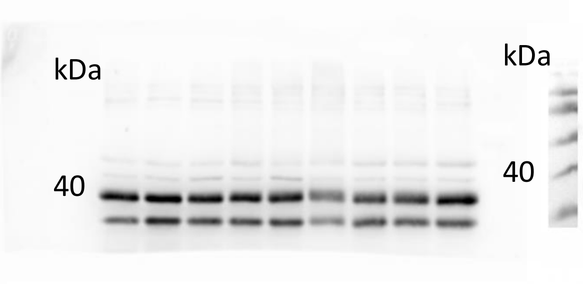

Figure 4 Panel C

P-4E-BP1 0, 6, 9, 12 h

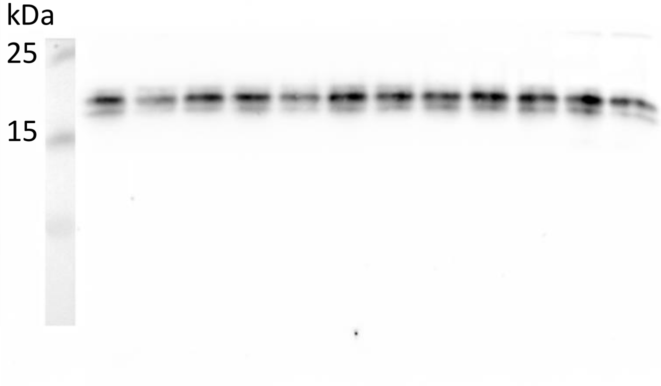

P-4E-BP1 0, 16,20 h

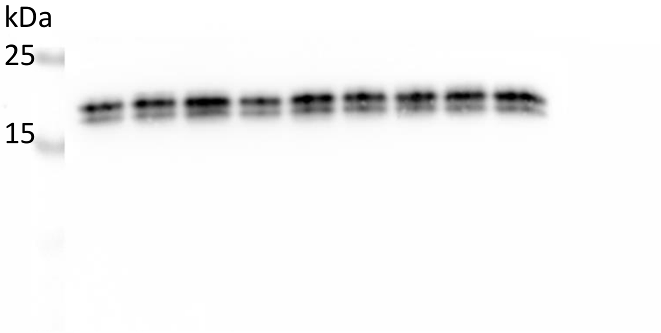

Gapdh 0, 6, 9, 12 h

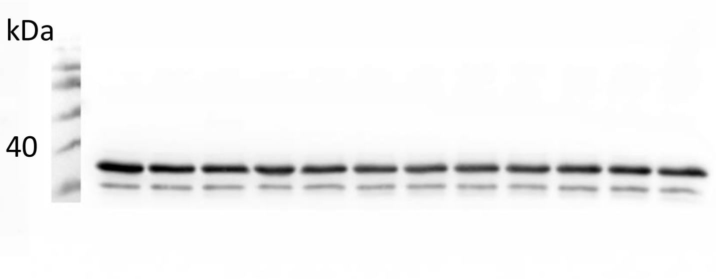

Gapdh 0,16,20 h

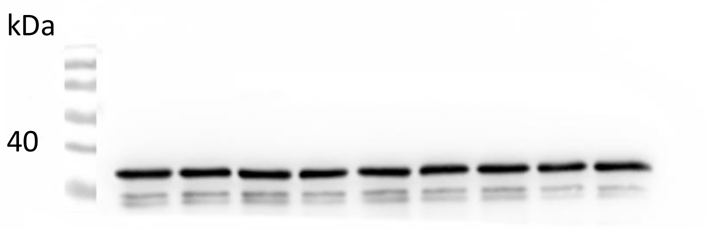

Figure 4 Panel C

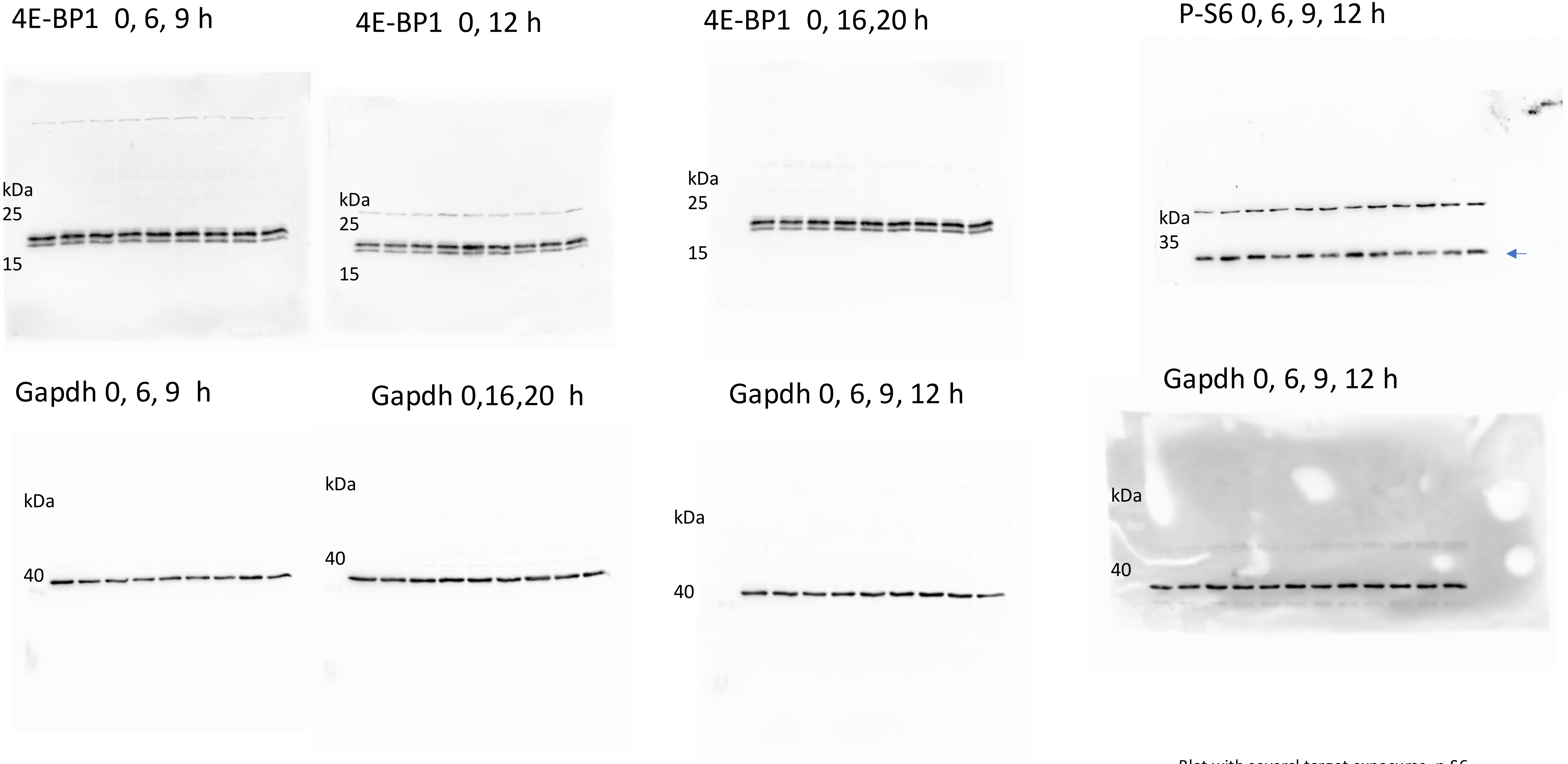

Last three lanes of blot are additional samples not displayed in this publication

Blot with several target exposures, p-S6 band marked with arrow

Figure 4 Panel C

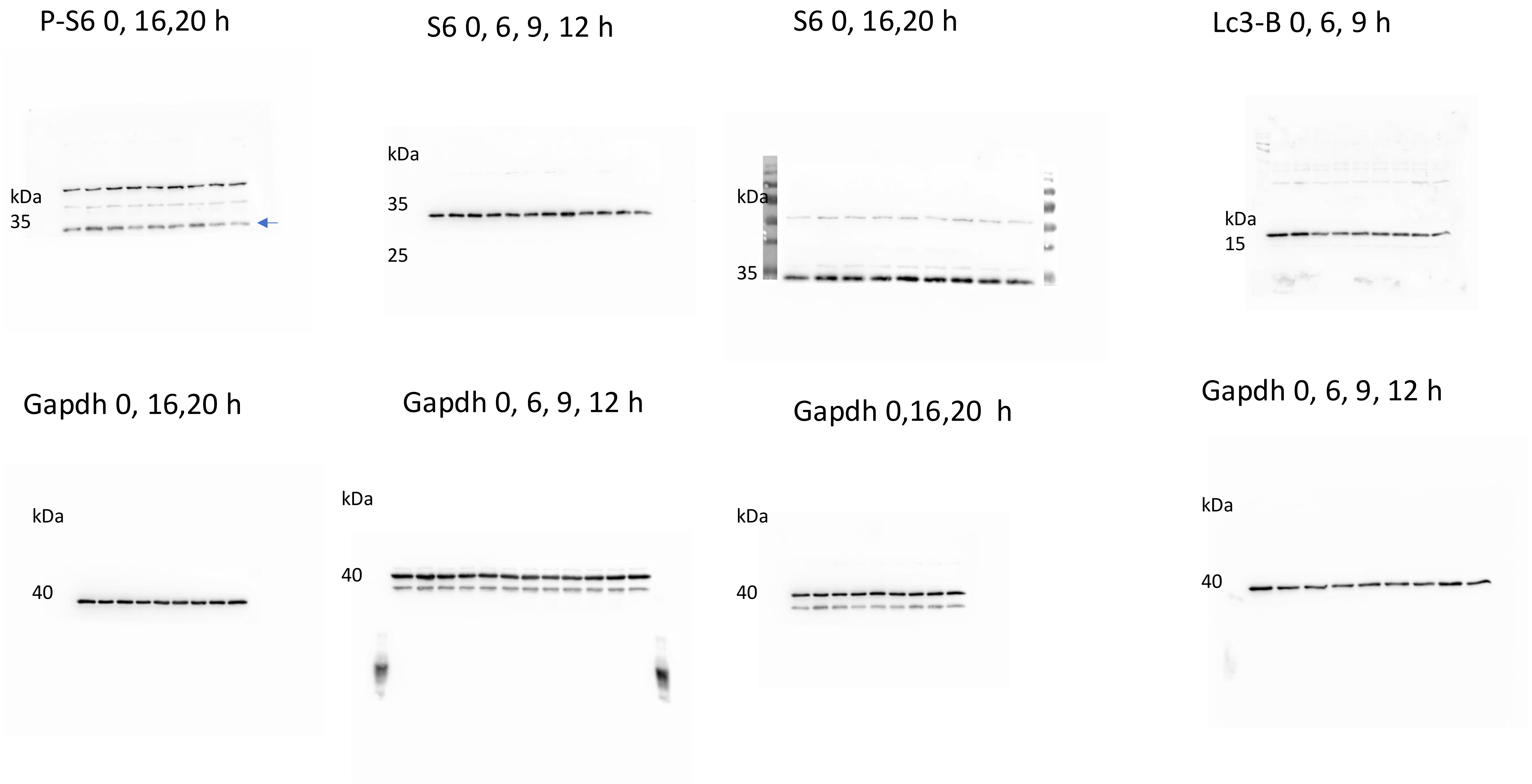

Blot with several target exposures, p-S6 band marked with arrow

Figure 4 Panel C

Lc3-B 0, 12 h

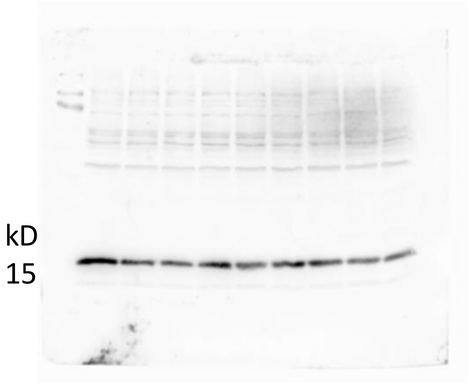

Lc3-B 0, 16, 20 h

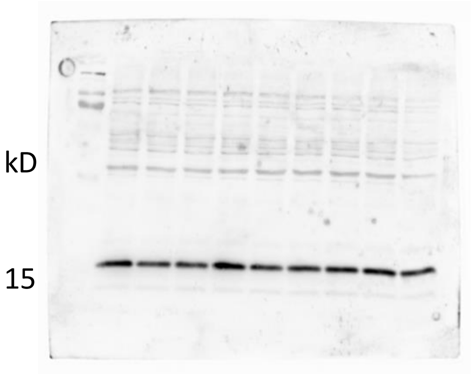

Gapdh 0, 12 h

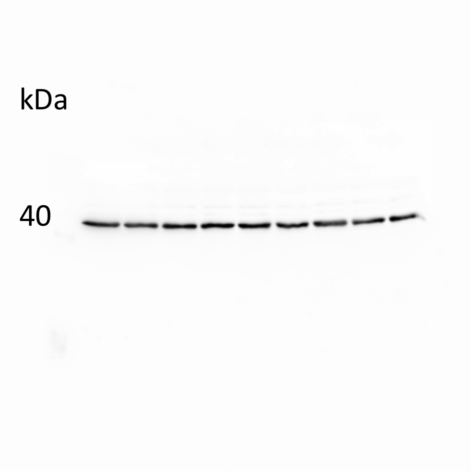

Gapdh 0, 16, 20 h

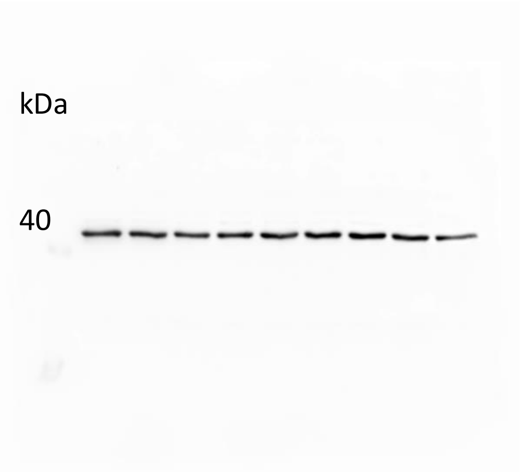

Last three lanes of blot are additional samples not displayed in this publication

Figure 4 Panel D

P-4E-BP1 0,6,9,12 h

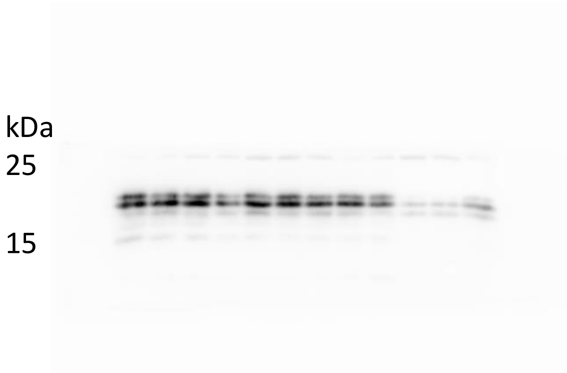

P-4E-BP1 0,16,20 h

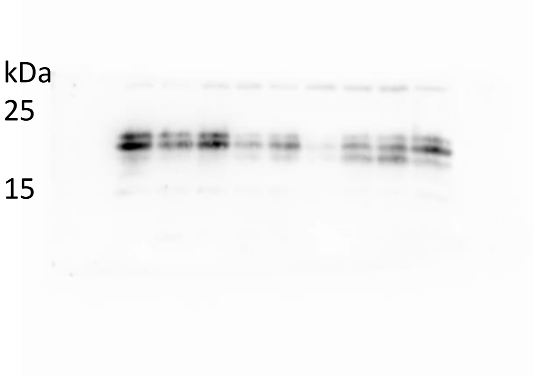

Gapdh 0, 6, 9, 12 h

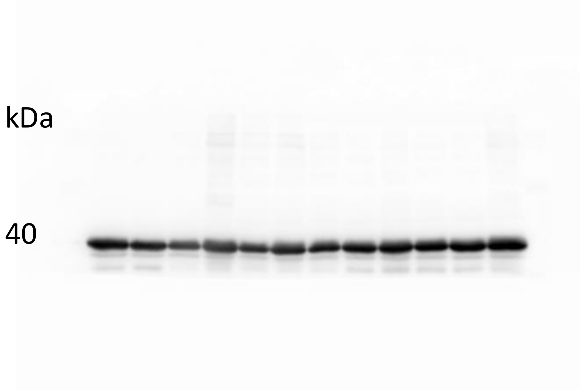

Gapdh 0, 16, 20 h

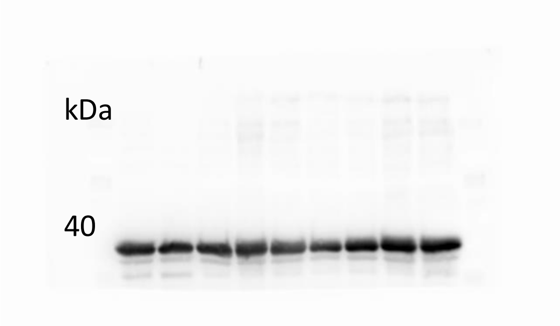

Figure 4 Panel D

4E-BP1 0,6,9 h

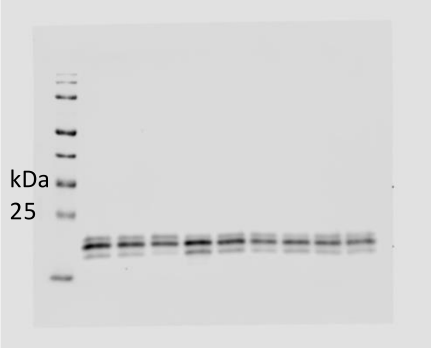

4E-BP1 0, 12 h

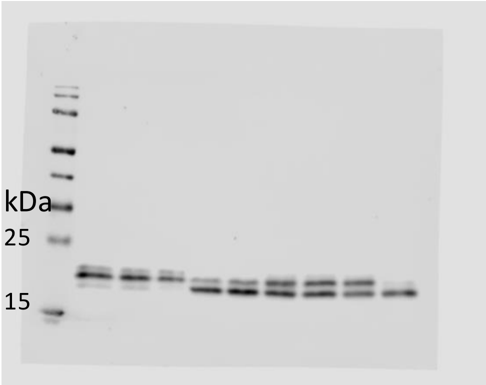

4E-BP1 0,16,20 h

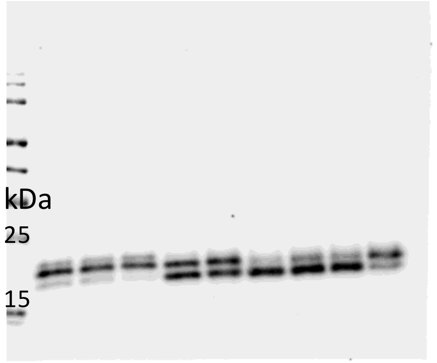

P-S6 0, 6, 9, 12 h

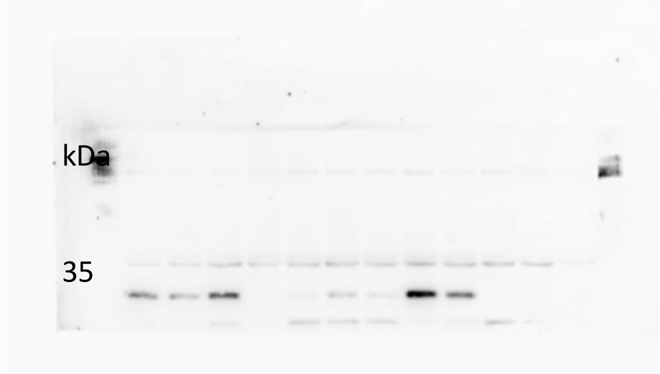

Gapdh 0,6,9 h

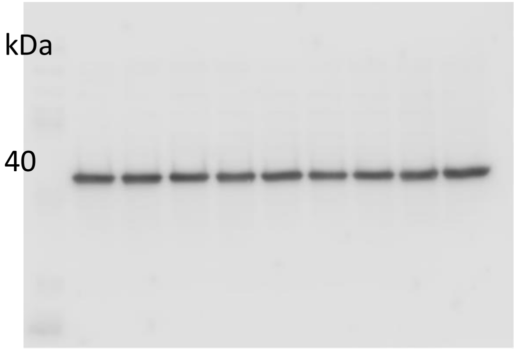

Gapdh 0, 12 h

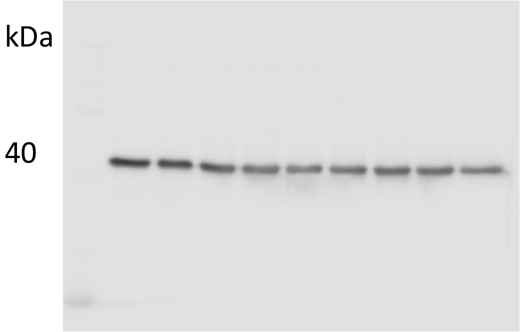

Gapdh 0, 16,20 h

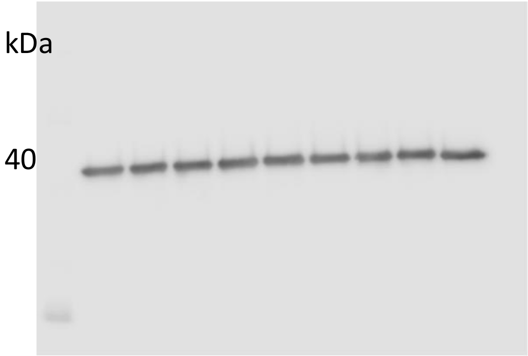

Gapdh 0,6,9,12 h

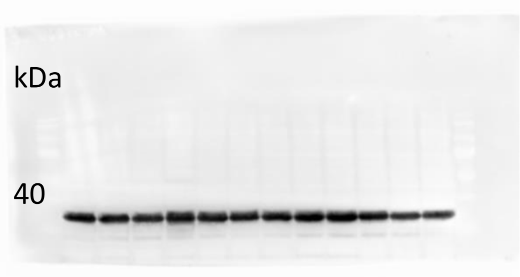

Figure 4 Panel D

P-S6 0, 16,20 h

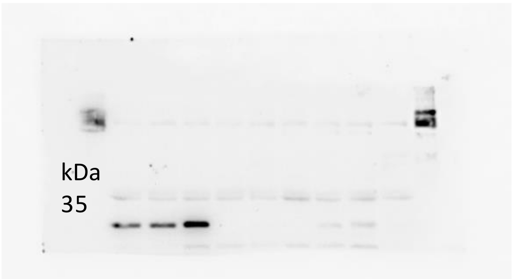

S6 0, 6,9,12 h

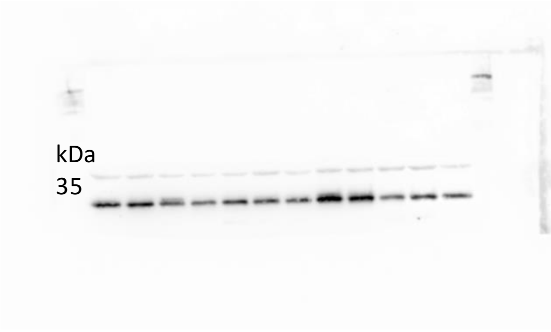

S6 0, 16,20 h

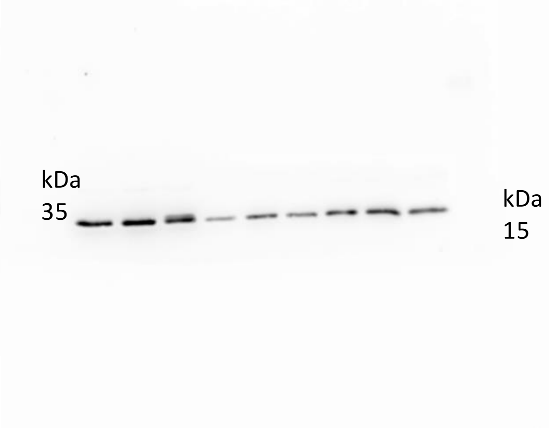

Lc3-B 0, 6, 9, 12 h

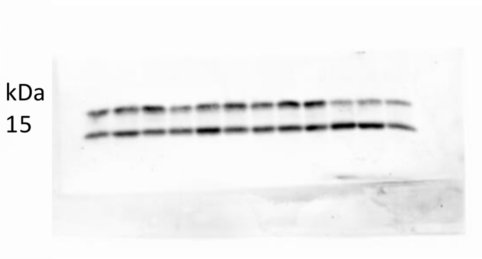

Lc3-B 0, 16, 20h

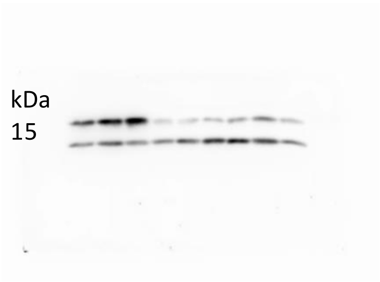

Gapdh 0,16,20 h

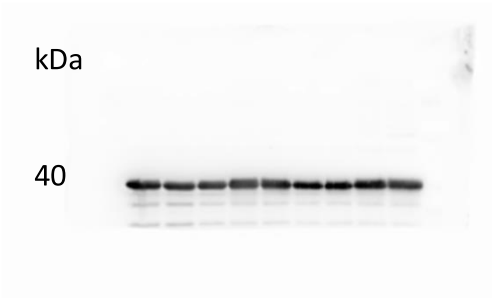

Gapdh 0,6,9, 12 h

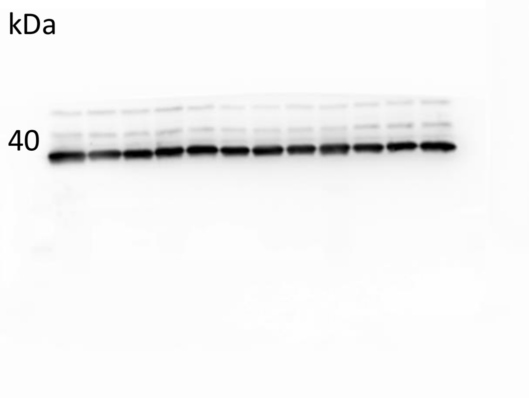

Gapdh 0, 16,20 h

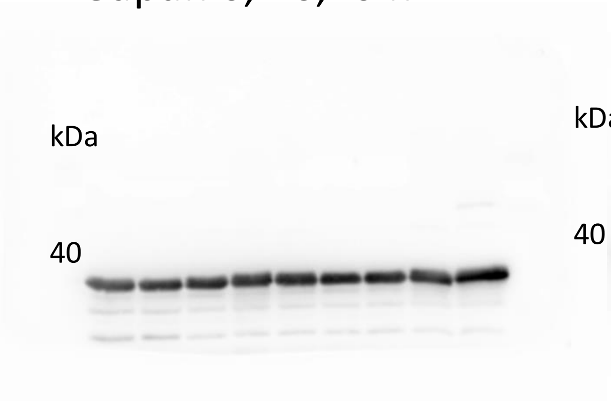

Gapdh 0,6,9, 12 h

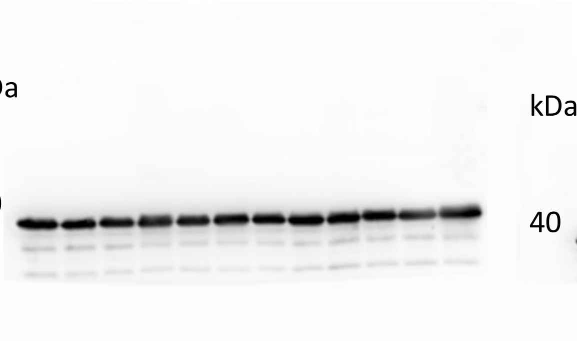

Gapdh 0, 16,20 h

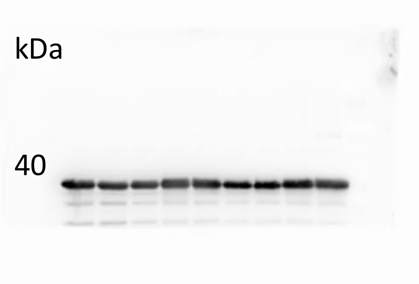

Figure 5 Panel A

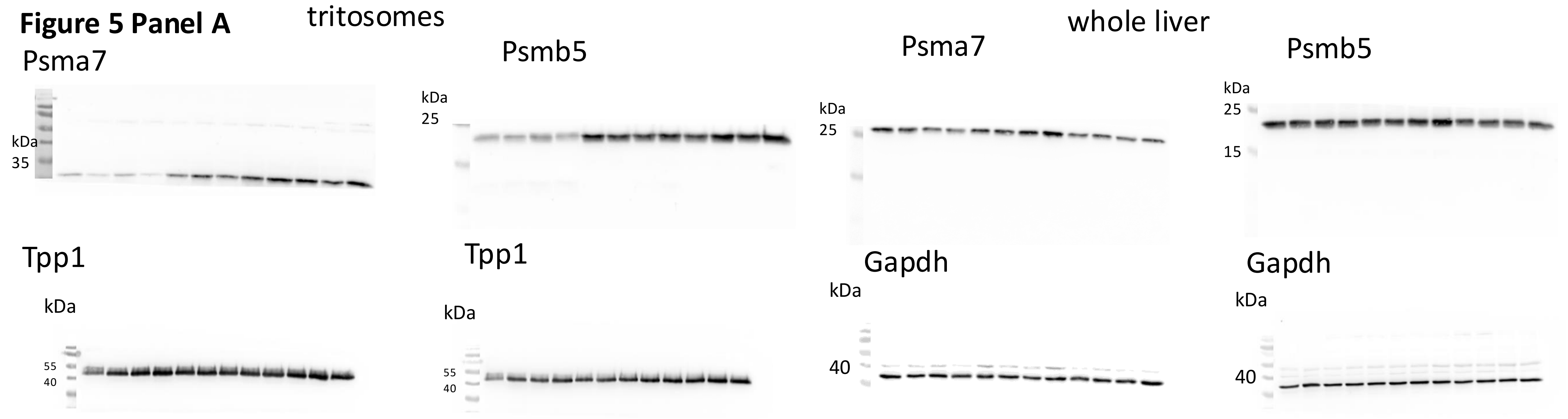

Figure 5 Panel B

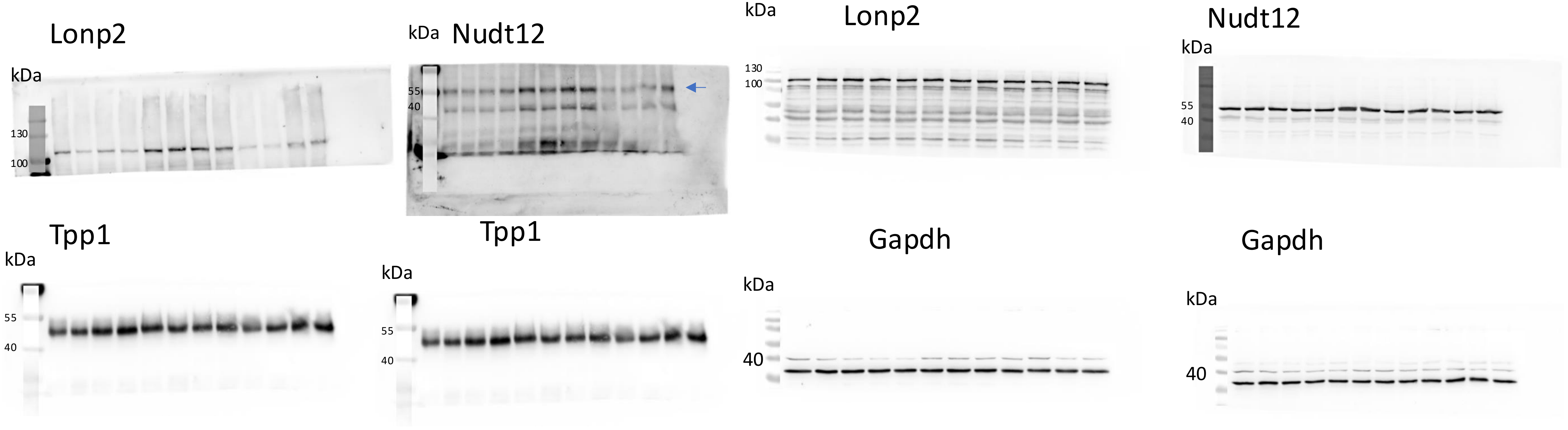

Note: Lonp2 and Nudt 12 were probed on the same membrane. Nudt marked by arrow due to previous signals.

Figure 5 Panel C

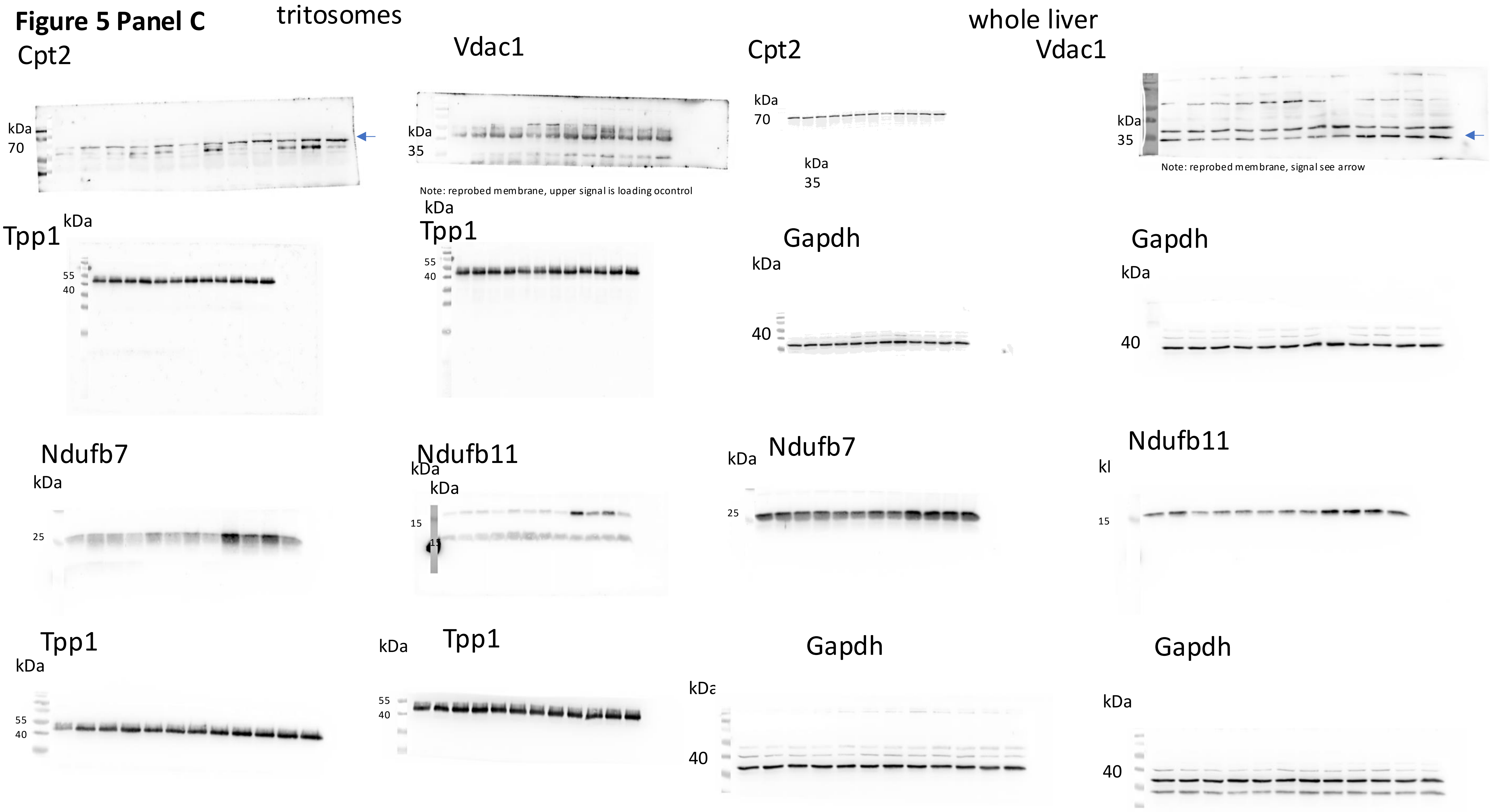

Supplementary Figure 1

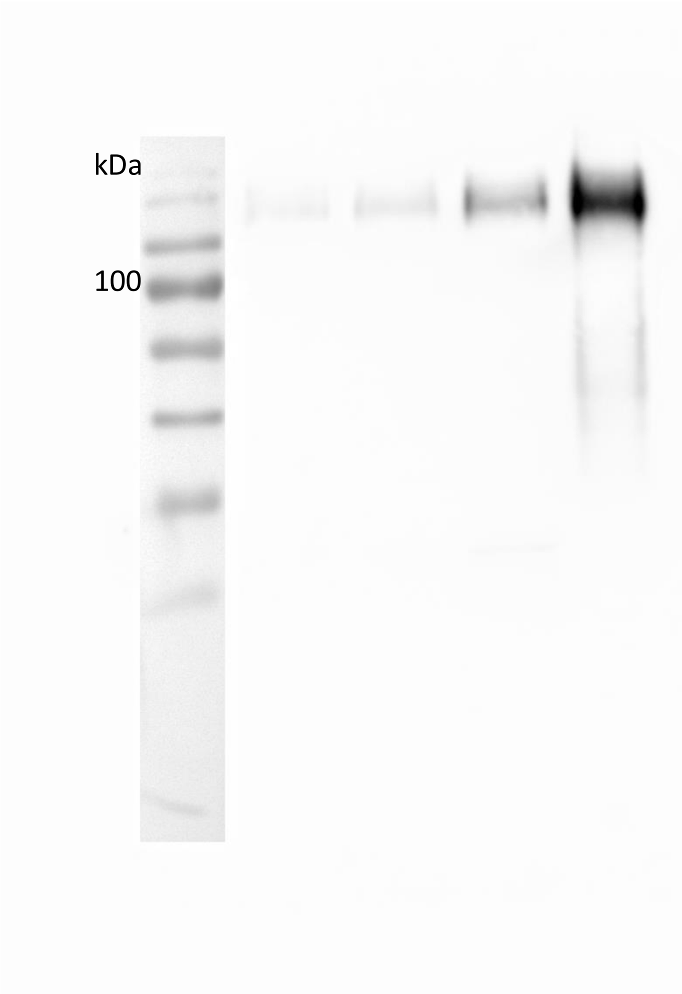

Lamp 1 0h

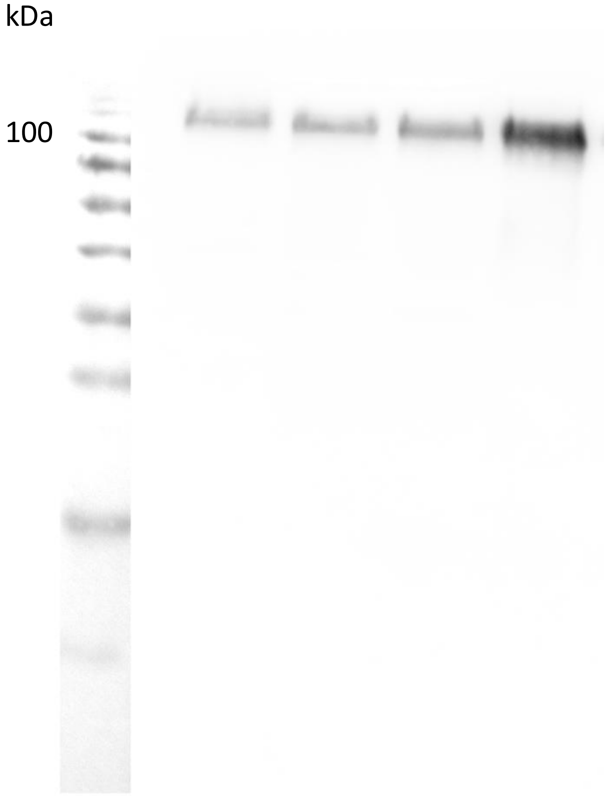

Lamp1 6h

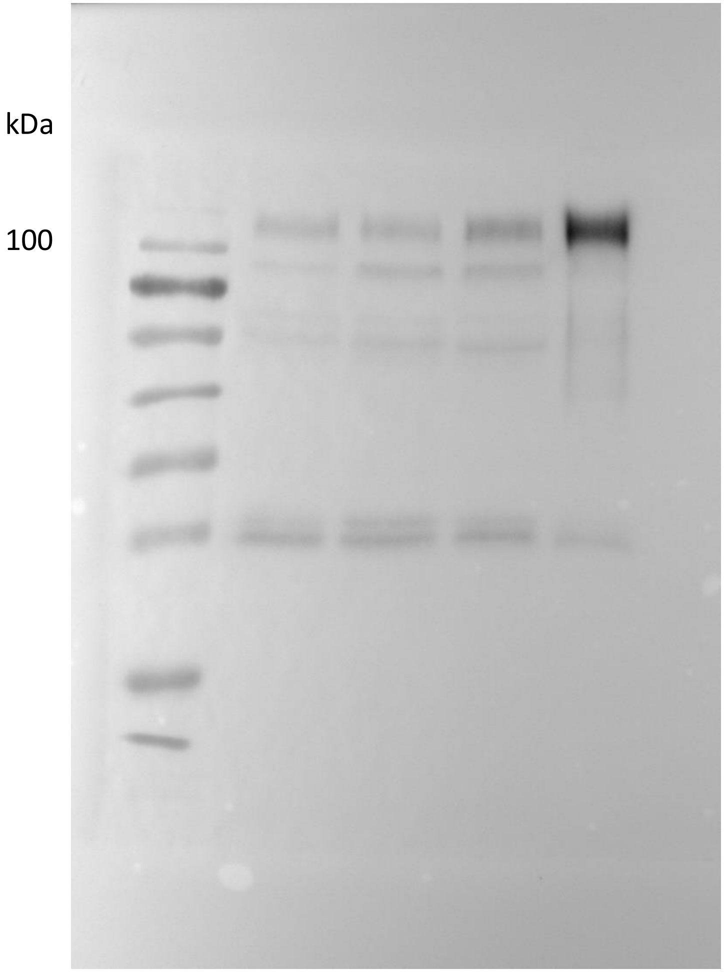

Lamp 1 24 h
